# Mamba-Bi-LSTM with SHAP-Guided Iterative Refinement for Multimodal ARDS Diagnosis: A Dual-System Framework

**DOI:** 10.3390/bioengineering13070794

**Published:** 2026-07-10

**Authors:** Mufeng Chen, Fuchang Luo, Jia Xie, Quansheng Ren

**Affiliations:** 1 Department of Engineering Science, University of Oxford, Parks Road, Oxford OX1 3PJ, UK; mufeng.chen@lmh.ox.ac.uk; 2 School of Electronics, Peking University, Science Building No. 5, Yiheyuan Lu, Haidian District, Beijing 100871, China; 2301213334@pku.edu.cn (F.L.); 2401213419@stu.pku.edu.cn (J.X.)

**Keywords:** acute respiratory distress syndrome, Mamba-Bi-LSTM, dual-system framework, multimodal fusion, SHAP-guided refinement, feature selection gate, EEG neurophysiology, early warning, ICU decision support

## Abstract

Acute respiratory distress syndrome (ARDS) is associated with mortality rates up to 46% and remains challenging to diagnose early due to overlapping clinical presentations. We propose a dual-system framework for multimodal ARDS diagnosis that integrates a Mamba-Bi-LSTM primary discrimination system with a TreeSHAP-based verification system whose attribution outputs iteratively refine the primary system’s feature selection gate. The primary system processes heterogeneous clinical inputs—ventilator parameters, blood gas indices, chest imaging, and EEG signals—through a selective state-space Mamba module and bidirectional LSTM layers. The verification system applies TreeSHAP attribution to independently cross-validate primary outputs, provide clinically interpretable evidence, and supply $\ell_{1}$-normalised attribution vectors that directly modulate the Mamba feature selection gate weights during offline refinement. A confidence-and-consistency decision mechanism governs final output, and high-confidence predictions are incorporated as curriculum-filtered signals to iteratively recalibrate both systems through a confidence-gated offline refinement protocol. Evaluated on 3742 held-out patients from MIMIC-IV (internal test) and 2594 patients from the eICU Collaborative Research Database across 208 US hospitals (external validation), the complete system achieves 92.8% accuracy and an F1 score of 0.889 after offline iterative recalibration on the internal test set, with 91.6% accuracy and F1 of 0.871 on external validation, extending early warning time from 5.2 to 9.7 h. The P/F ratio consistently ranks as the top predictive feature in alignment with the Berlin definition. Ablation experiments confirm that EEG integration independently contributes a 2.7 percentage point accuracy gain and a 1.9-h extension of the warning window (McNemar $\chi^{2}=27.0$, p<0.001). All performance improvements over single-modality baselines and over existing methods are statistically significant (p<0.001, Bonferroni-corrected). End-to-end processing latency of 350 ms per case is compatible with real-time ICU deployment.

## 1. Introduction

Acute respiratory distress syndrome (ARDS) is a critical illness, and its clinical diagnosis faces severe challenges.

First, early identification is difficult. The early symptoms of ARDS are similar to those of many respiratory diseases, such as common pneumonia and cardiogenic pulmonary edema, which are difficult to distinguish clinically. According to the Berlin definition [1], the diagnosis of ARDS must meet multiple criteria, including acute onset, bilateral lung infiltration, and abnormal oxygenation index (${\mathrm{PaO}}_{2}/{\mathrm{FiO}}_{2}$), but early manifestations are often atypical or incomplete. Studies have shown that the global misdiagnosis rate of ARDS is as high as 40%, resulting in a large number of patients failing to receive appropriate treatment in time. In community hospitals and primary medical institutions, the recognition rate of ARDS is even lower due to the lack of professional equipment and specialists.

Secondly, the diagnosis of ARDS requires comprehensive analysis of multi-dimensional medical data, which increases the complexity of diagnosis. Physicians need to simultaneously evaluate the patient’s clinical symptoms, ventilator parameters, blood gas analysis results, chest imaging changes, and other indicators, and conduct dynamic monitoring. This multi-dimensional evaluation places extremely high demands on the professional quality and clinical experience of physicians, especially in the early stages of the disease, when the changes in various indicators may not be obvious, which increases the difficulty of judgment.

Third, ARDS progresses rapidly and has a high mortality rate. Studies have shown that the mortality rate of ARDS increases with the severity of the disease, and the mortality rate of severe ARDS is as high as 46%. The disease can progress rapidly within a few hours, and missing the critical treatment window will lead to irreversible prognosis. However, traditional monitoring methods often make it difficult to achieve continuous real-time monitoring of multiple physiological indicators, resulting in physicians being unable to detect the trend of disease changes in time and missing the opportunity for intervention.

In addition, the individualisation of ARDS treatment plans also faces challenges. Patients vary significantly in their response to treatment, and treatment strategies need to be dynamically adjusted according to individual conditions, including ventilator parameter settings, fluid management, prone ventilation, etc. This highly individualised treatment adjustment requires physicians to have rich experience and accurate judgment, which is particularly difficult when medical resources are limited.

In this paper, we introduce a dual-system diagnostic framework for ARDS that concurrently optimises diagnostic accuracy, computational efficiency, multimodal data integration, and clinical interpretability. The primary discrimination component is grounded in a Mamba-Bi-LSTM architecture augmented with an inter-channel EEG spatial correlation module specifically designed for the neurological monitoring demands of ICU patients. An independent verification component, employing clinically interpretable feature engineering and TreeSHAP attribution within a random forest classifier, evaluates the same inputs in parallel without sharing learned representations with the primary system. Concordance and confidence thresholds jointly govern the final clinical alert, and predictions exceeding the high-confidence criterion—subject to dual-system agreement—are used to iteratively recalibrate each subsystem’s parameters through a confidence-gated offline refinement protocol.

The main contributions of this work are as follows:

(i) Hybrid temporal architecture with empirical justification. We propose a Mamba-Bi-LSTM hybrid architecture for multimodal ARDS diagnosis, achieving 92.8% accuracy on 3742 held-out patients. The hybrid design is empirically justified through ablation: the Mamba-only variant (89.2% accuracy, F1 = 0.863) and Bi-LSTM-only variant (88.1%, F1 = 0.857) each underperform the hybrid by 3.6 and 4.7 percentage points respectively, demonstrating that Mamba’s selective state-space long-range modelling and Bi-LSTM’s bidirectional contextual encoding are complementary rather than redundant. Supplementary EEG-derived neurophysiological features are incorporated for the EEG-available training subset (n=320), with claims regarding EEG contribution restricted to this population.
(ii) Multimodal database construction and temporally aligned preprocessing. We integrate heterogeneous clinical data streams—ventilator parameters (high-frequency, ∼1 Hz), blood gas indices (intermittent, 2–6 h intervals), chest imaging (daily), and EEG (continuous waveform)—from MIMIC-IV, MIMIC-III, and eICU-CRD, with systematic modality-specific preprocessing and a common-reference temporal alignment protocol ensuring cross-modal time consistency.
(iii) Dual-system architecture with active SHAP-guided refinement. The SHAP-based verification module operates as a structurally independent diagnostic tier that actively intervenes in output determination: $\ell_{1}$-normalised TreeSHAP attribution vectors are fed back into the Mamba feature-selection gate, directly modulating gate weights toward clinically validated features. This design distinguishes the verification system from conventional post-hoc explanation tools and provides clinicians with transparent, auditable diagnostic reasoning aligned with Berlin definition criteria.
(iv) Robust missing-modality adaptation and rigorous external validation. A missing-modality adaptation mechanism redistributes modality-level weighting when any input stream is absent; ablation across four modality-availability configurations (with and without EEG, with and without chest imaging) confirms that the mechanism consistently reduces the performance gap by 2.7–4.3 percentage points relative to naive masking. External validation on 2594 eICU patients across 208 US hospitals (91.6% accuracy) demonstrates strong cross-institutional generalisation, with all reported improvements statistically significant (p<0.001, Bonferroni-corrected McNemar’s test).

## 2. Related Work

Machine learning and deep learning have demonstrated sustained progress in ICU clinical decision support. Churpek et al. established that ML methods consistently outperform conventional regression for predicting in-hospital clinical deterioration using routinely collected physiological and laboratory variables [2]. Building on this, Ruiz et al. showed that integrating electronic health record variables including vital signs and laboratory measurements enables prediction of postoperative deterioration hours in advance, illustrating the broader potential of data-driven approaches for timely ICU intervention [3]. These results collectively motivate the development of more comprehensive, multimodal diagnostic frameworks tailored to high-acuity conditions such as ARDS.

ARDS-specific prediction has attracted growing research attention, with several approaches establishing important performance benchmarks. Deep learning methods combining temporal clinical signals with imaging have shown particular promise: Meyer et al. demonstrated that LSTM-CNN architectures can predict ICU complications in real time [4], while Jabbour et al. showed that fusing chest radiographs with electronic health record data improves diagnosis of acute respiratory failure [5]. For ARDS early warning specifically, Singhal et al. developed and validated eARDS—an interpretable ML algorithm applied to 35,804 patients across multiple institutions—achieving AUROC of 0.89 and enabling detection up to 12 h prior to Berlin criteria onset [6]. A 2025 systematic review and meta-analysis of 33 studies reported an overall pooled AUC of 0.91 (0.88–0.93) for AI-based ARDS prediction; however, single-modality configurations showed substantially lower performance—mechanical ventilation parameters alone yielded AUC 0.87 (0.83–0.89) and non-imaging, non-laboratory predictors yielded AUC 0.78 (0.74–0.81)—while multimodal integration of images with other predictors achieved the best AUC of 0.92 (0.89–0.94), confirming the performance advantage of richer multimodal frameworks [7].

The Mamba selective state-space model [8] has recently emerged as a strong alternative to Transformer-based architectures for long clinical time-series, offering linear computational complexity without sacrificing representational capacity. APRICOT-Mamba [9] applied a Mamba-based network to ICU acuity prediction across 107,473 patients from 55 hospitals, demonstrating practical advantages over attention-based models in handling sparse, irregularly sampled clinical sequences. Building on our prior work applying the Mamba-Bi-LSTM and SHAP verification paradigm to epilepsy diagnosis [10], the present study extends this dual-system framework to the more complex and clinically urgent ARDS setting, scaling evaluation to a substantially larger and geographically diverse patient cohort.

Interpretability has become a prerequisite for clinical adoption of AI systems. The Foundation Model Transparency Index [11] highlights transparency as a core requirement for trustworthy deployment. Model-level attribution tools such as SHAP and LIME [12] provide theoretically grounded feature-level explanations, enabling clinicians to audit individual predictions. In the present work, the SHAP-based verification system functions not merely as a post-hoc explanation module but as a structurally independent diagnostic component that actively cross-validates the primary system’s outputs—a design choice that meaningfully distinguishes this framework from prior interpretability-augmented approaches.

## 3. Methodology

All methods described in this study were performed in accordance with the relevant guidelines and regulations. The research protocols followed the Declaration of Helsinki principles for human research ethics. Data collection and processing procedures adhered to the Health Insurance Portability and Accountability Act (HIPAA) privacy guidelines for de-identified health information. Machine learning model development followed the Transparent Reporting of a multivariable prediction model for Individual Prognosis or Diagnosis (TRIPOD) guidelines. Clinical data handling protocols were implemented according to Good Clinical Practice (GCP) standards, and all computational methods were validated following the FDA Software as a Medical Device (SaMD) Action Plan (U.S. Food & Drug Administration) recommendations where applicable.

### 3.1. Dataset

To ensure that the system has sufficient learning samples and generalisation capabilities, this study integrated multiple internationally recognised critical care databases. The primary modalities comprise ventilator parameters, arterial blood gas indices, chest imaging, and laboratory measurements—consistent with the Berlin definition [1]. For a subset of MIMIC-III patients with available waveform records, supplementary electroencephalogram (EEG) data were incorporated to explore the potential of neurophysiological monitoring in capturing brain–lung interactions that conventional respiratory parameters cannot reflect [13].

The MIMIC-IV (v2.0), MIMIC-III (v1.4), and eICU Collaborative Research databases were accessed for research purposes between November 2024 and June 2025. No author had access to information that could identify individual participants during or after data collection, as all records were fully de-identified prior to access.

#### 3.1.1. Data Source

The MIMIC-IV v2.0 database served as the primary data source for this study. The database contains de-identified health data from Beth Israel Deaconess Medical Center in the United States from 2001 to 2019. This study screened 36,390 ICU patients from the database, of whom 24,947 additionally met the Berlin definition for ARDS [1] and were included as ARDS cases. Of the 36,390 patients, 18,895 died in hospital and 17,495 survived. Patient demographic and clinical variables extracted from the database encompassed baseline characteristics (hospitalization timestamps, ICU admission duration, mortality outcomes where applicable, patient demographics including gender and age), continuous physiological monitoring parameters (cardiac rhythm metrics, pulmonary function indicators, core body temperature, haemodynamic pressures including systolic/diastolic measurements and calculated mean arterial pressure), established severity assessment tools (Sequential Organ Failure Assessment, Simplified Acute Physiology Score II, Charlson Comorbidity Index), and comprehensive laboratory biomarkers (hematological profiles including leukocyte enumeration, haemoglobin concentration, haematocrit percentage, platelet quantification, acid-base balance indicators, renal function markers, electrolyte panels, glucose metabolism parameters, coagulation cascade assessments, arterial blood gas analysis including pH and oxygenation status). This standardised data extraction methodology follows the MIMIC-IV database schema described by Johnson et al. [14] and aligns with critical care research data harmonisation protocols established by Pollard et al. [15].

The eICU Collaborative Research Database (eICU-CRD) [15] served as the independent external validation dataset. This multi-centre database comprises deidentified health data from over 200,000 ICU admissions across 208 hospitals and 335 units in the United States (2014–2015), collected via the Philips eICU telehealth programme. From this database, 2594 ICU patients were identified, of whom 847 (32.6%) were classified as ARDS-positive and 1747 as non-ARDS controls. Because eICU-CRD does not provide standardised chest radiograph records, ARDS labels were operationalised using Berlin-derived physiological surrogates: (i) PaO_2_/FiO_2_ ≤ 300 mmHg, (ii) PEEP ≥ 5 cmH_2_O, and (iii) invasive mechanical ventilation or CPAP support. This surrogate labelling approach is consistent with established practice for applying Berlin-equivalent criteria to databases lacking structured imaging [1]. Available data include vital sign measurements, ventilator parameters, arterial blood gas indices, laboratory measurements, and APACHE severity scores. As eICU-CRD provides neither continuous EEG monitoring nor standardised chest imaging, external validation employed two modalities: ventilator parameters and blood gas indices, supplemented by laboratory measurements and APACHE severity scores. This reduced-modality configuration was handled by the model’s missing-modality adaptation mechanism, which masks absent input channels and redistributes modality-level weighting across the remaining feature streams.

**Figure 1:**
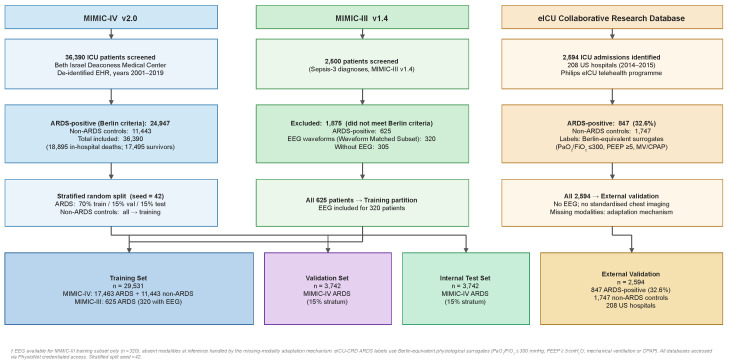
Patient inclusion and exclusion flow diagram across all three data sources. MIMIC-IV (v2.0): 36,390 ICU patients screened; 24,947 ARDS-positive (Berlin Definition criteria); 11,443 non-ARDS controls; stratified random split (seed = 42): 70% training/15% validation/15% test (ARDS patients); non-ARDS controls allocated to training only. MIMIC-III (v1.4): 2500 patients screened (Sepsis-3 diagnoses); 1875 excluded (did not meet Berlin Definition criteria); 625 ARDS-positive, of whom 320 had EEG waveform data (MIMIC-III Waveform Matched Subset); all assigned to the training partition only. eICU Collaborative Research Database: 2594 ICU patients identified (208 US hospitals, 2014–2015); 847 ARDS-positive (32.6%); 1747 non-ARDS controls; ARDS labels operationalised using Berlin-equivalent physiological surrogates (PaO_2_/FiO_2_ ≤ 300 mmHg, PEEP ≥ 5 cmH_2_O, mechanical ventilation or CPAP); all reserved for external validation only. Final datasets: Training (n=29,531: MIMIC-IV 17,463 ARDS + 11,443 non-ARDS; MIMIC-III 625 ARDS including 320 with EEG ^†^); Validation (n=3742, MIMIC-IV ARDS, 15% stratum); Internal Test Set (n=3742, MIMIC-IV ARDS, 15% stratum); External Validation (n=2594, eICU-CRD, no EEG, no standardised imaging). ^†^ EEG available for MIMIC-III training subset only; absent modalities handled by the missing-modality adaptation mechanism at inference.

The MIMIC-III v1.4 database served as an additional training source. From this database, 2500 patients with Sepsis-3 diagnoses were screened, of whom 625 met the Berlin definition criteria for ARDS. The same data extraction schema and preprocessing pipeline described above for MIMIC-IV were applied to MIMIC-III, with one exception: MIMIC-III does not include the MIMIC-CXR chest radiograph linkage, so imaging features for these patients were derived solely from structured radiology report text rather than raw image data. EEG data for 320 of these patients were obtained by cross-referencing subject identifiers with the MIMIC-III Waveform Database Matched Subset (a companion resource hosted on PhysioNet that provides bedside physiological waveforms, including multichannel EEG, for a subset of MIMIC-III subjects with verified clinical record linkage), applying the same electrode-system standards and preprocessing pipeline described in Section 3.1.3. All MIMIC-III patients were assigned to the training split only (70% partition) and were not used in validation or testing, ensuring that the external test cohort remained independent of this auxiliary source. Figure 1 presents the patient inclusion and exclusion flow diagram across all three data sources.

#### 3.1.2. EEG as Supplementary Neurophysiological Signal

EEG data were incorporated as a supplementary neurophysiological modality for the subset of MIMIC-III patients with available waveform records (n=320, all assigned to the training split). The physiological rationale for EEG integration in ARDS rests on three converging lines of evidence.

##### Brain–Lung Bidirectional Interaction

The interaction between the brain and lungs in critically ill patients is bidirectional and well-documented [16,17]. ARDS patients frequently manifest neurological sequelae: over 80% of ARDS survivors experience secondary neurological disorders including cognitive impairment, post-traumatic stress disorder, depression, and anxiety, and approximately 50% face enduring neurocognitive impairments two years after ICU discharge [17,18]. The underlying mechanisms include hypoxia-induced cerebral dysfunction, systemic neuroinflammation, ventilator-associated brain injury (characterised by neuroinflammation and increased neuronal apoptosis), and blood-brain barrier disruption [16]. These mechanisms produce measurable EEG signatures—including background slowing, frontal rhythmic delta activity, and loss of reactivity to external stimuli—that are detectable before clinical neurological deterioration becomes apparent [19,20].

##### EEG Abnormalities in ARDS and COVID-19-Related ARDS

Severe ARDS, particularly COVID-19-related ARDS, is frequently accompanied by diffuse encephalopathy, ICU delirium, cortical slowing, and hypoxia-related cerebral dysfunction [13,19]. Electroencephalographic studies of COVID-19 ICU patients report abnormal EEG findings in 69–96% of cases, with generalised background slowing present in approximately 60% [20,21]. Characteristic patterns include theta-predominant background slowing (4–8 Hz), frontal intermittent rhythmic delta activity (FIRDA), and non-reactive monomorphic EEG—all indicative of diffuse cortical involvement and metabolic encephalopathy [19]. These EEG features correlate with clinical severity, duration of mechanical ventilation, and long-term neuropsychological outcomes [16,18], providing prognostic information that conventional respiratory and haemodynamic parameters cannot capture.

##### Scope and Limitations of EEG Integration

Importantly, EEG is not positioned here as a primary diagnostic biomarker for ARDS, nor as a replacement for Berlin definition criteria. Rather, EEG-derived neurophysiological features serve as a complementary signal that enriches the model’s representation of systemic critical illness in patients where continuous neurological monitoring is already available—a subpopulation that includes severe ARDS, prolonged sedation, unexplained encephalopathy, and COVID-related neurological complications. The ablation results quantify EEG’s incremental contribution within this training subset. Because EEG monitoring is not universally available across ICU settings—as reflected by its absence in the eICU external validation cohort—the system incorporates a missing-modality adaptation mechanism (Section 4.6) that masks the EEG input and re-weights remaining feature streams, ensuring robust performance regardless of EEG availability. Claims regarding the diagnostic value of EEG (+2.7 percentage points accuracy, +1.9 h warning time) are restricted to the EEG-available training subpopulation (n=320, MIMIC-III) and should not be generalised to the broader ARDS patient population without prospective validation in a dedicated EEG-monitored cohort.

#### 3.1.3. Data Structure and Processing

In the process of integrating these datasets, this study first conducted a data consistency check, performed multiple interpolation on missing data, detected outliers and retained data within the range of 1–99%. To handle the differences between datasets, the research team standardised all variable values to ensure the comparability of data from different sources. For continuous variables that exhibit nonlinear relationships (such as heart rate, mean arterial pressure, respiratory rate, body temperature, pH, urine volume, and blood glucose), the research team converted them into categorical variables based on their data distribution type and clinical significance. The experimental dataset composition is summarised in Table 1.

To ensure data quality, this study adopted a strict data cleaning process, completely removing variables with more than 20% missing observations, and using multiple interpolation methods for variables with a missing rate of less than 20%. At the same time, the research team used VIM and mice packages to visualise and process missing values to ensure the integrity and reliability of the data.

To avoid data leakage between the training, validation, and test sets, the research team applied a stratified 70%/15%/15% random split to the 24,947 ARDS-positive MIMIC-IV patients, yielding an internal test set of 3742 cases. Non-ARDS control patients from MIMIC-IV were allocated to training only, and the eICU-CRD cohort was reserved entirely as an independent external validation set. The random split was performed with a fixed seed (seed = 42) to ensure reproducibility. All data preprocessing steps—including imputation, normalisation, and feature extraction—were fitted exclusively on the training partition and applied without refitting to the validation and test partitions, ensuring no information leakage.

##### Data Leakage Control in Iterative Refinement

The validation partition $D_{\mathrm{val}}$ serves a dual purpose in the offline refinement protocol (Algorithm 1): it is used to construct the high-confidence refinement pool $R_{t}$ and to monitor AUC for early stopping. Crucially, the held-out test set $D_{\mathrm{test}}$ (the 3742 MIMIC-IV patients) was never accessed during any phase of iterative refinement—it was used solely for final performance reporting after all refinement cycles were completed. No model parameters, decision thresholds, or feature selection rules were tuned using test-set information. The role of $D_{\mathrm{val}}$ in refinement is analogous to a development/tuning set in conventional hyperparameter optimisation; all reported test-set metrics are therefore free of feedback-induced bias. This design is explicitly encoded in Algorithm 1 (Steps 4–11), which operates exclusively on $D_{\mathrm{train}}\cup D_{\mathrm{val}}$.

The preprocessing of EEG data includes multiple steps: first, baseline correction and bandpass filtering (0.5–70 Hz) are performed to remove environmental noise and motion artifacts; second, eye movement and electromyographic artifacts are removed; and then time domain, frequency domain, and time-frequency domain features are extracted. In view of the differences in different monitoring devices and sampling rates, we developed a unified data standardisation process to ensure data consistency and comparability.

To handle the high-dimensional characteristics of EEG data, we used an automated algorithm to extract key features, including: (1) background continuity score, (2) background reactivity index, (3) frequency band power ratio, (4) absolute and relative power of each frequency band (δ, θ, α, β), (5) periodic and rhythmic pattern detection results, and (6) frontal EEG asymmetry index. These EEG features, together with traditional clinical indicators, constitute the input data of the ARDS intelligent diagnosis system.

In the process of data integration, we paid special attention to the time synchronisation of EEG data with other physiological signals, and used an automatic alignment algorithm based on a common reference signal to ensure the time consistency of the data, laying the foundation for multimodal data fusion.

##### Temporal Alignment Across Heterogeneous Modalities

The four input modalities operate at substantially different sampling rates and acquisition schedules. Ventilator parameters are recorded continuously at approximately 1 Hz; arterial blood gas indices are collected intermittently at 2–6 h clinical intervals; chest radiographs are acquired once daily or on clinical indication; and EEG waveforms are streamed continuously at 256 Hz before feature extraction. To achieve cross-modal temporal consistency, all modality streams are aligned to a common 1-h epoch grid anchored to ICU admission time. Ventilator parameters are aggregated within each epoch using five-number summary statistics (minimum, 25th, median, 75th, maximum) to preserve physiological variation. Blood gas values are interpolated onto the epoch grid using cubic spline interpolation (Equation (3)); epochs without any blood gas measurement within a ±3 h window are flagged and handled by the missing-data imputation protocol described below. Chest imaging features are propagated forward to subsequent epochs until a new image is acquired, consistent with the clinical reality that radiographic status is considered stable until the next examination. EEG features are computed over 30-s windows and then aggregated per epoch.
**Algorithm 1** Offline Confidence-Gated Iterative Refinement**Require:** 
Training partition $D_{\mathrm{train}}$, validation partition $D_{\mathrm{val}}$; initial primary system $\theta_{P}$, verification system $\theta_{V}$; high-confidence threshold $\tau_{h}=0.92$; feedback scaling factor λ (empirically determined on $D_{\mathrm{val}}$); maximum iterations T=4; early stopping tolerance ε=0.001**Ensure:** 
Refined parameters $\theta_{P}^{*}$, $\theta_{V}^{*}$ 1:Pre-train $\theta_{P}$ on $D_{\mathrm{train}}$ (Adam optimiser, cosine annealing, early stop on validation $F_{1}$) 2:Pre-train $\theta_{V}$ on $D_{\mathrm{train}}$ (stratified 5-fold cross-validation) 3:**for** t=1 **to** *T* **do** 4:    Run fixed-weight inference with $\theta_{P}$ and $\theta_{V}$ over $D_{\mathrm{val}}$; compute S(x) via Equation (19) for each *x* 5:    **Construct refinement pool** (curriculum gate):         $R_{t}\leftarrow \left\{x\in D_{\mathrm{val}}:S\left(x\right)>\tau_{h}\right\}$▹ Equivalent to dual-agreement; see Equation (19) with β=0.6 6:    All cases with $S\left(x\right)\leq \tau_{h}$ excluded from $R_{t}$; disagreement cases routed for clinician review 7:    Compute TreeSHAP attributions over $R_{t}$; aggregate and $\ell_{1}$-normalise per feature to obtain $\Phi_{t}\in R^{F}$ 8:    **FSG modulation** via Equation (20): $W_{g,t}^{\prime }\leftarrow W_{g}⊙\left(1+\lambda \;1_{H}^{⊤}⊗\Phi_{t}\right)$; row-wise $\ell_{2}$-normalise 9:   Freeze Mamba SSM and Bi-LSTM backbone; fine-tune FSG parameters ($W_{g,t}^{\prime }$) and classification head of $\theta_{P}$ on $D_{\mathrm{train}}\cup R_{t}$ (ground-truth labels)10:    Retrain $\theta_{V}$ on $D_{\mathrm{train}}\cup R_{t}$ (ground-truth labels)11:    Evaluate ${\mathrm{AUC}}_{t}$ of $\theta_{P}$ on $D_{\mathrm{val}}$12:    **if** t>1 **and** ${\mathrm{AUC}}_{t}-{\mathrm{AUC}}_{t-1}<\varepsilon$ **then**13:        **break** ▹ Early stopping: marginal AUC gain below convergence threshold14:    **end if**15:**end for**16:**return** $\theta_{P}^{*}\leftarrow \theta_{P}^{\left(t\right)}$,$\theta_{V}^{*}\leftarrow \theta_{V}^{\left(t\right)}$

##### Missing Data Handling

Within each modality, variables with a missing rate exceeding 20% are excluded; variables with a missing rate below 20% are imputed using multivariate imputation by chained equations (MICE) [3], applied independently within each data partition (training, validation, test) to prevent information leakage across splits. For the missing-modality case—where an entire input channel (e.g., chest imaging or EEG) is absent for a patient encounter—the missing-modality adaptation mechanism masks the corresponding input channel and redistributes modality-level weights across the remaining feature streams via the modality-adaptive weighting layer described in Section 3.3.1. The quantitative impact of this mechanism is evaluated in Section 4.6.

### 3.2. Multimodal Data Preprocessing

The performance of the ARDS intelligent diagnosis system depends on the quality of multimodal data, including ventilator parameters, blood gas analysis indicators, and chest imaging data. The collection and processing of ARDS data focuses more on the real-time nature of dynamic physiological monitoring and imaging characteristics [2] to adapt to the acute progression of the disease.

#### 3.2.1. Ventilator Parameter Processing

In terms of ventilator parameter processing, data mainly comes from the patient’s respiratory support equipment, reflecting the dynamic changes of respiratory function. The collected parameters include tidal volume (TV), positive end-expiratory pressure (PEEP), peak airway pressure ($P_{\mathrm{peak}}$) and respiratory rate (RR), which are usually recorded at high frequency (several to dozens of times per minute). In order to eliminate noise, a median filter is used to smooth the original signal:

(1) $$S_{\mathrm{smooth}}\left(t\right)=\mathrm{median}\left\{S\left(t-i\right)|i=-k,\ldots ,k\right\}$$

Among them, S(t) represents the original signal value at a certain time point, *k* is the window radius (usually 5), and $S_{\mathrm{smooth}}\left(t\right)$ is the smoothed signal value. In order to extract trend features, the local weighted regression (LOESS) method [22] is further applied to fit the parameter change curve:

(2) $$S_{\mathrm{trend}}\left(t\right)=\sum_{i}w_{i}\left(t\right)\;S\left(t_{i}\right)$$

Among them, $w_{i}\left(t\right)$ is a weighted function based on time distance, emphasising the contribution of neighbouring points. Outlier detection combines clinical threshold ($P_{\mathrm{peak}}>40\;{\mathrm{cmH}}_{2}O$) and Z-score method to mark points that exceed 3 times the standard deviation. Short-term missing (less than 5 s) is repaired by linear interpolation, and long-term missing is completed using an autoregressive model to ensure data continuity.

#### 3.2.2. Blood Gas Analysis Index Processing

The processing of blood gas analysis indicators is aimed at the patient’s oxygenation and acid-base balance. Key indicators include arterial oxygen partial pressure (${\mathrm{PaO}}_{2}$), arterial carbon dioxide partial pressure (${\mathrm{PaCO}}_{2}$), pH value, and lactate concentration. Since these data are collected intermittently and have poor time consistency, they need to be interpolated based on the blood collection time point, using the cubic spline interpolation method:

(3) $$S\left(t\right)=at^{3}+bt^{2}+ct+d$$

Among them, *a*, *b*, *c*, *d* are coefficients fitted according to adjacent blood sampling points, and S(t) is the index value after interpolation. Quality control sets the physiological range (such as ${\mathrm{PaO}}_{2}$ within a clinically plausible range of 20–600 mmHg, which accommodates both hypoxaemic patients and those receiving high-flow supplemental oxygen—a range that is essential for preserving the integrity of the P/F ratio feature central to ARDS diagnosis), and abnormal values outside this range are supplemented or eliminated after manual verification. The oxygenation index (P/F ratio) is used as a comprehensive indicator:

(4) $$P/F=\frac{{\mathrm{PaO}}_{2}}{{\mathrm{FiO}}_{2}}$$

Among them, ${\mathrm{FiO}}_{2}$ is the inspired oxygen fraction (expressed as a decimal, e.g., 0.21 for room air), and the P/F ratio is used to evaluate the lung oxygenation capacity, which forms a unified feature set after alignment with the ventilator parameters.

#### 3.2.3. Image Data Feature Extraction

The feature extraction of chest image data focuses on lung pathological changes, such as ground-glass opacity and consolidation. This pipeline was applied to MIMIC-IV patients, for whom raw chest radiograph images are available via MIMIC-CXR linkage, and to MIMIC-III patients, for whom imaging features were derived from structured radiology report text. For the eICU external validation cohort, chest imaging features were excluded entirely, as eICU-CRD does not provide standardised radiograph records; the missing-modality adaptation mechanism compensated for this absence. Preprocessing first enhances the contrast through histogram equalisation:

(5) $$H^{\prime }\left(I\right)=\frac{\mathrm{cdf}\left(I\right)-{\mathrm{cdf}}_{\min}}{{\mathrm{cdf}}_{\max}-{\mathrm{cdf}}_{\min}}\cdot \left(L-1\right)$$

where $H^{\prime }\left(I\right)$ is the pixel value after equalisation, cdf is the cumulative distribution function, and *L* is the grayscale level (the value used in this study is 256). Subsequently, the U-Net model was used for lung segmentation [23] with a segmentation accuracy of 96%, extracting the texture features (such as contrast and uniformity) and morphological features (such as area and boundary complexity) of the lesion area. To support time series analysis, the rate of change between consecutive images was calculated by the difference method:

(6) $$\Delta I=|I_{t+1}-I_{t}|$$

where $I_{t}$ and $I_{t+1}$ are image data at adjacent time points, and ΔI reflects the progression rate of the lesion. The processed image features are fused with physiological data to form a multimodal input.

### 3.3. Main Discrimination System Construction

The primary discrimination system is the architectural centrepiece of the ARDS diagnosis framework. Its principal function is to ingest heterogeneous ICU data streams and produce a continuous severity assessment across the four-class ARDS spectrum (Normal, Mild, Moderate, Severe).

#### 3.3.1. Mamba-Bi-LSTM Hybrid Architecture

##### Rationale for the Hybrid Design

ICU monitoring data for ARDS diagnosis presents two distinct temporal challenges that no single architecture handles optimally. First, physiological time series—particularly ventilator waveforms and EEG—span tens of hours and require efficient long-range sequence modelling with sub-quadratic complexity; the Mamba selective state-space model [8] addresses this through input-dependent state selection, achieving linear scaling without the memory cost of full attention. Second, ARDS onset is characterised by bidirectional contextual cues: future deterioration patterns (e.g., worsening P/F ratio trajectories) are as informative as past trends, and retrospective context is essential for distinguishing ARDS from cardiogenic pulmonary oedema with similar early presentations; bidirectional LSTM [24] captures this bidirectional dependency through its forward and backward hidden state concatenation. The two architectures therefore address complementary modelling requirements: Mamba provides computationally efficient long-horizon global context, while Bi-LSTM provides fine-grained bidirectional local context.

This complementarity is empirically confirmed by ablation (Section 4.2): the Mamba-only variant achieves 89.2% accuracy (F1 = 0.863, warning time 9.2 h) and the Bi-LSTM-only variant achieves 88.1% (F1 = 0.857, 8.8 h), while the hybrid achieves 92.8% (F1 = 0.889, 9.7 h)—a gain of 3.6 and 4.7 percentage points over the respective single-architecture baselines. The hybrid also outperforms both architectures on warning time, indicating that the long-range modelling strengths of Mamba and the bidirectional contextual strengths of Bi-LSTM jointly contribute to earlier detection.

**Figure 2:**
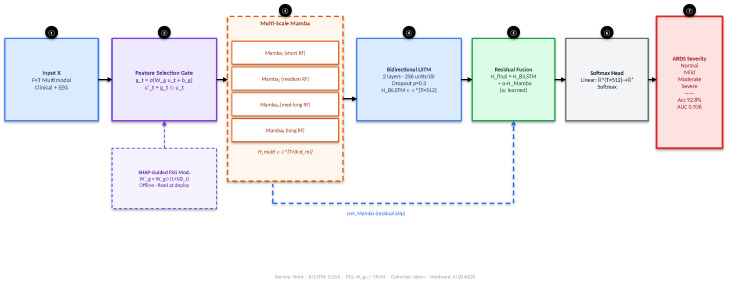
Mamba-Bi-LSTM hybrid architecture design.

An end-to-end learning paradigm governs the primary discrimination system, whose computational backbone is a Mamba-Bi-LSTM hybrid network designed to process heterogeneous ICU data streams without manual feature engineering. Discriminative representations are learned directly from raw multimodal inputs, allowing the system to uncover latent patterns spanning ventilator parameters, blood gas indices, imaging features, and EEG signals simultaneously. Four functional blocks constitute this pipeline: a multimodal ingestion layer, a modality-specific feature extraction layer, the Mamba-Bi-LSTM hybrid processing core, and a severity classification output layer; the hybrid processing core is the architecturally defining component, whose structural design is illustrated in Figure 2.

The Mamba architecture is a selective state-space model (SSM) parameterised by four learned matrices: $A\in R^{N\times N}$ (state transition), $B\in R^{N\times 1}$ (input projection), $C\in R^{1\times N}$ (output projection), and Δ∈R (timescale parameter). Unlike prior SSMs with fixed parameters, Mamba computes Δ, *B*, and *C* as functions of the input sequence, enabling selective retention of relevant context. The discretisation uses zero-order hold: $\bar{A}=\exp\left(\Delta A\right)$ and $\bar{B}={\left(\Delta A\right)}^{-1}\left(\bar{A}-I\right)\Delta B$. The hidden state dimension *N* was set to 64 in this study, balancing representational capacity with computational efficiency for the multimodal clinical input sequences.

In the hybrid architecture of this study, the Mamba layer first processes the input sequence and then feeds its output into the Bi-LSTM layer for bidirectional processing:

(7) $$H_{\mathrm{Mamba}}=\mathrm{Mamba}\left(X\right)$$

Three targeted modifications to the baseline Mamba architecture were introduced to address the heterogeneity and long-horizon nature of ICU monitoring data in ARDS:

1. Selective feature gating: The standard Mamba state-update is augmented with a feature selection gate (FSG) that suppresses non-discriminative activations before the state transition, directing model capacity toward clinically relevant patterns in ARDS monitoring data:
  (8) $$g_{t}=\sigma \left(W_{g}u_{t}+b_{g}\right)$$
  (9) $$u_{t}^{\prime }=g_{t}⊙u_{t}$$
2. Residual skip pathways: Vanishing-gradient instability is mitigated by introducing residual connections that bridge the Mamba and Bi-LSTM sub-networks, permitting stable backpropagation through the full hybrid depth:
  (10) $$H_{\mathrm{final}}=H_{\mathrm{Bi}-\mathrm{LSTM}}+\alpha \;H_{\mathrm{Mamba}}$$
3. Parallel multi-resolution processing: A multi-scale Mamba module applies several parallel Mamba blocks with distinct receptive field widths, subsequently merging their outputs so that both short-interval physiological fluctuations and long-horizon trend patterns characteristic of ARDS progression are captured simultaneously:
  (11) $$H_{\mathrm{multi}}=\mathrm{Concat}\;\left({\mathrm{Mamba}}_{1}\left(X\right),\;{\mathrm{Mamba}}_{2}\left(X\right),\;\ldots ,\;{\mathrm{Mamba}}_{k}\left(X\right)\right)$$

A two-phase curriculum guided model optimisation: the Mamba and Bi-LSTM sub-networks were pre-trained independently to establish stable initial parameter configurations, after which the integrated architecture was jointly fine-tuned end-to-end. This staged protocol substantially reduces gradient conflicts arising from the structural heterogeneity of the hybrid network and was found to accelerate convergence relative to random initialisation of the joint model.

All experiments were implemented in PyTorch (v2.0) on a single NVIDIA A100 40 GB GPU (CUDA 11.8, 40 GB HBM2 memory). The primary discrimination system was trained using the Adam optimiser with an initial learning rate of $1\times 10^{-4}$, weight decay of $1\times 10^{-5}$, and a cosine annealing schedule decaying to $1\times 10^{-6}$ over 100 training epochs. Batch size was set to 32. Gradient clipping with a maximum norm of 1.0 was applied to stabilise training of the Mamba–Bi-LSTM hybrid. Early stopping was triggered after 10 consecutive epochs without improvement in validation F1 score. The Mamba hidden state dimension *N* was set to 64; the Bi-LSTM used 2 bidirectional layers with 256 hidden units per direction. Dropout (p=0.3) was applied between layers. The random forest verification system comprised 500 trees with a maximum depth of 20 and minimum samples per leaf of 5, trained with stratified 5-fold cross-validation. All reported computation times were measured on the same A100 hardware to ensure comparability.

##### Reproducibility Details

All experiments used fixed random seeds to ensure deterministic behaviour: random.seed(42) (Python 3.9), np.random.seed(42) (NumPy 1.24.3), torch.manual_seed(42) (PyTorch v2.0), and torch.cuda.manual_seed_all(42) (CUDA 11.8). The data split identifiers (patient subject IDs assigned to each partition) and the MIMIC variable item IDs used for data extraction are available upon request to the corresponding author. The hyperparameter search space for the primary system covered learning rates in $\left\{10^{-3},10^{-4},10^{-5}\right\}$, hidden dimensions in {32,64,128}, dropout rates in {0.1,0.2,0.3}, and batch sizes in {16,32,64}; the selected configuration was determined by validation AUC. For the random forest verification system, the grid search covered number of trees in {100,200,500}, maximum depth in {10,15,20}, and minimum samples per leaf in {2,5,10}. All following comparisons used the same held-out test partition and preprocessing pipeline to ensure fair evaluation; differences in AUC are accompanied by 95% confidence intervals estimated via 1000-sample bootstrapping, and pairwise AUC comparisons are assessed using the DeLong test [25]. Software dependencies: scikit-learn 1.3.0, SHAP 0.42.1, numpy 1.24.3, pandas 2.0.3.

#### 3.3.2. Inter-Channel Spatial Correlation Processing

Beyond their temporal content, multi-channel EEG recordings encode spatially distributed patterns of cortical activity that carry substantial diagnostic and prognostic value in ARDS. Synchronisation dynamics between brain regions are perturbed by the hypoxaemic and metabolic derangements characteristic of ARDS, and capturing these inter-regional relationships supplements the temporal representations extracted by the Mamba-Bi-LSTM processing core.

According to the electrode positions of the international 10–20 system as standardised by Jasper [26], this study defines an adjacency matrix $A\in R^{N\times N}$, where *N* is the number of EEG channels and $A_{i,j}$ represents the anatomical connection relationship between channel *i* and channel *j*. This adjacency matrix not only takes into account the physical distance between electrodes, but also refers to known functional connectivity patterns of brain regions, such as the connection between the frontal lobe and the temporal lobe, which is of special significance in ARDS-related brain lesions, especially for capturing the common frontal lobe EEG abnormal patterns in COVID-19 patients.

A Graph Mamba Network (GMN) was constructed around this adjacency matrix to jointly model inter-channel spatial relationships and temporal dynamics. GMN treats each EEG electrode as a graph node and integrates spectral graph convolution [27] with Mamba sequence modelling, enabling simultaneous propagation of neighbourhood-level spatial information and temporal state-space representations across the full electrode montage:

(12) $$X^{\left(l+1\right)}=\sigma \;\left({\tilde{D}}^{-\frac{1}{2}}\tilde{A}\;{\tilde{D}}^{-\frac{1}{2}}X^{\left(l\right)}W^{\left(l\right)}\right)$$

(13) $$H^{\left(l+1\right)}=\mathrm{Mamba}\;\left(X^{\left(l+1\right)}\right)$$

where $\tilde{A}=A+I_{N}$ is the adjacency matrix with self-connection added, $\tilde{D}$ is the degree matrix of $\tilde{A}$, $W^{\left(l\right)}$ is the learnable weight matrix of the *l*-th layer, and σ is a nonlinear activation function.

This design enables the model to consider both spatial topological structure and temporal evolution patterns, which is crucial for identifying common diffuse and regional EEG abnormalities in ARDS patients. Studies have shown that the EEG of ARDS patients can present different degrees of background slowing, frontal rhythmic activity, and non-reactive patterns, which are closely related to clinical prognosis.

**Figure 3:**
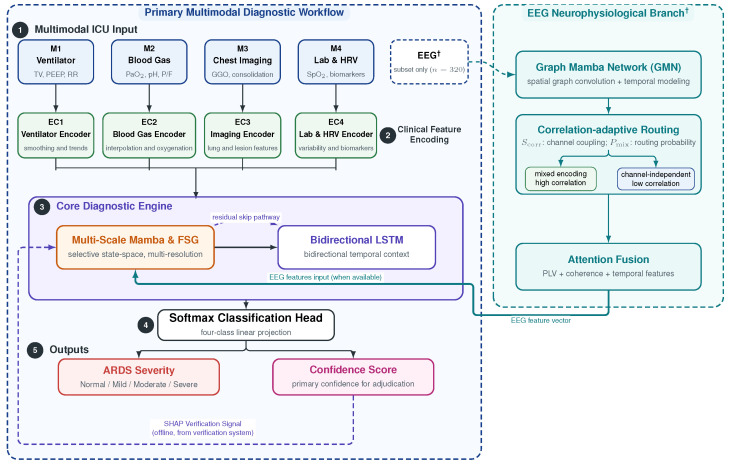
Workflow of the primary discrimination system. ^†^EEG neurophysiological branch available for MIMIC-III training subset only (n=320); absent modalities handled by the missing-modality adaptation mechanism.

Figure 3 illustrates the complete workflow of the primary discrimination system.

A correlation-adaptive routing switch steers EEG feature flows through either channel-independent or channel-mixed processing pathways depending on the instantaneous inter-electrode correlation structure. A scalar correlation index $S_{\mathrm{corr}}$ is computed from the current EEG segment and mapped via a learned gating function to a mixing probability $P_{\mathrm{mix}}$, governing the information routing decision:

(14) $$S_{\mathrm{corr}}=\frac{1}{N(N-1)}\sum_{i\neq j}\left|\mathrm{Corr}(X_{i},X_{j})\right|$$

(15) $$P_{\mathrm{mix}}=\sigma \;\left(W_{\mathrm{switch}}\;S_{\mathrm{corr}}+b_{\mathrm{switch}}\right)$$

$S_{\mathrm{corr}}$ quantifies aggregate pairwise inter-channel coupling in the current segment, and $P_{\mathrm{mix}}$ determines routing probability toward the mixed-encoding branch. Highly correlated electrode configurations—indicative of global cortical synchronisation such as that encountered in metabolic encephalopathy or diffuse ARDS-related brain dysfunction—preferentially activate the mixed-encoding path, while recordings with weaker inter-channel coupling are routed through independent channel encoders that are more sensitive to focal abnormalities.

In particular, in patients with ARDS, this mechanism is of particular value because it can distinguish global brain dysfunction (such as metabolic encephalopathy) from local dysfunction (such as ischaemic changes or microbleeds), both of which may occur in patients with severe ARDS.

In order to more comprehensively capture the dynamic interactions between brain regions, this study calculated the following two spatial features:

- Phase Locking Value (PLV): PLV quantifies the temporal consistency of instantaneous phase differences between electrode pairs across successive measurement windows, serving as a model-free marker of functional connectivity strength between cortical regions. It is computed as:
  (16) $${\mathrm{PLV}}_{xy}=\left|\frac{1}{N}\sum_{j=1}^{N}e^{\;i\left(\phi_{x}\left(j\right)-\phi_{y}\left(j\right)\right)}\right|$$
- Spectral coherence between channel pairs: Coherence characterises the frequency-resolved linear dependence between channels, revealing band-specific synchronisation patterns that PLV alone does not resolve. It is defined as:
  (17) $$C_{xy}\left(f\right)=\frac{{\left|S_{xy}\left(f\right)\right|}^{2}}{S_{xx}\left(f\right)\;S_{yy}\left(f\right)}$$
  Here $S_{xy}\left(f\right)$ denotes the cross-spectral density between channels *x* and *y*, while $S_{xx}\left(f\right)$ and $S_{yy}\left(f\right)$ are the respective autospectral densities. Coherence adds frequency-specificity relative to PLV, making the two metrics complementary in characterising the diverse EEG abnormalities observed across ARDS subtypes and disease stages.

An attention module dynamically re-weights PLV, coherence, and temporal feature streams prior to fusion, accommodating the variable diagnostic relevance of each stream at different stages of ARDS progression. This multidimensional fusion approach meaningfully improved model sensitivity for ARDS-associated cortical dysfunction, providing more precise prognostic signals—particularly in COVID-19 ARDS, where early identification of frontal lobe EEG abnormalities is important for guiding clinical decision-making.

### 3.4. Verification System Construction

The validation system adopts a strategy combining feature engineering with traditional machine learning methods to extract features with clear clinical significance from multimodal data and analyse the contribution of features to diagnostic decisions through the SHAP (SHapley Additive exPlanations) method. Its workflow is different from the primary discrimination system based on the Mamba architecture.

**Figure 4:**
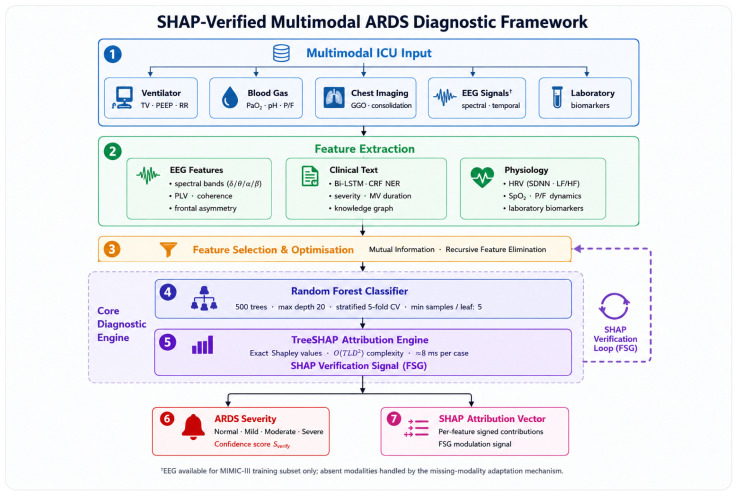
Verification system workflow.

Figure 4 shows the workflow of the SHAP-based verification system.

The verification system generates standalone ARDS severity assessments through its feature-engineering pipeline while simultaneously providing the cross-validation signal that the dual-system decision mechanism requires to grade diagnostic certainty and route ambiguous cases for clinician review.

#### 3.4.1. Multidimensional Feature Extraction

Multidimensional feature extraction serves to distil from the heterogeneous ICU data streams those signal characteristics most predictive of ARDS onset and severity. Because primary respiratory and haemodynamic parameters may not fully capture the neurological sequelae that influence ARDS prognosis, EEG-derived features are extracted with explicit attention to markers of cortical dysfunction, including spectral slowing, abnormal synchronisation, and non-reactive background patterns. Time-domain, frequency-domain, and time-frequency decomposition methods are combined to construct a comprehensive multi-resolution feature set, following the EEG feature engineering framework of Subasi [28] and its medical signal processing adaptations described by Acharya et al. [29]. Table 2 summarises the key features extracted from each data modality.

#### 3.4.2. Interpretability Analysis via SHAP Method

TreeSHAP attribution constitutes the interpretability engine of the verification system, assigning each clinical and EEG-derived feature a signed contribution value that quantifies its net influence on a given ARDS severity prediction. The implementation adheres to the unified Shapley-value framework formalised by Lundberg and Lee [30], with exact tree-structure exploitations detailed in the TreeSHAP extension by Lundberg et al. [31]. Shapley values—originating from cooperative game theory—provide a theoretically principled decomposition of the model output into per-feature contributions derived from their marginal coalition effects.

The calculation formula of the SHAP value is as follows:

(18) $$\phi_{i}=\sum_{S\subseteq N\setminus \{i\}}\frac{|S|!\;(|N|-|S|-1)!}{|N|!}\left[f(S\cup \{i\})-f(S)\right]$$

Here $\phi_{i}$ denotes the Shapley value of feature *i*, *N* is the complete feature set, *S* ranges over all subsets excluding feature *i*, and f(S) is the model output evaluated on subset *S*. The weighted sum enumerates all possible feature coalitions, ensuring a complete and equitable attribution decomposition. Two axiomatic properties underpin the clinical reliability of this framework: efficiency (all feature contributions sum to the difference between the prediction and the expected model output), and monotonicity (a feature whose inclusion consistently raises model outputs in any coalition receives a non-negative Shapley value).

Regarding computational cost, SHAP value calculation can be expensive in high-dimensional settings. In this study, the verification system employs a random forest classifier, which enables the use of TreeSHAP—an exact, polynomial-time algorithm that exploits the tree structure to compute Shapley values in $O(TLD^{2})$ time, where *T* is the number of trees, *L* is the maximum number of leaves, and *D* is the maximum tree depth. This is significantly more efficient than the exponential-time exact Shapley computation or the model-agnostic KernelSHAP approximation. In practice, TreeSHAP computes the full feature attribution vector for a single patient encounter in approximately 8 ms on the A100 GPU platform used in this study, well within the latency budget of the 350 ms end-to-end pipeline. For the complete 3742-sample test set, batch SHAP computation added approximately 30 s in total, representing less than 5% overhead relative to the main inference time.

A random forest classifier serves as the backbone of the verification system, operating on the engineered feature set to discriminate ARDS severity levels. Its ensemble-tree architecture is inherently more amenable to exact feature attribution than deep neural networks, enabling TreeSHAP to replace the exponentially complex exact Shapley computation with a polynomial-time algorithm.

In the diagnosis of ARDS, the application of the SHAP method has the following advantages:

- Its results directly correspond to indicators of clinical concern, such as EEG waveform features or oxygenation index, so that the interpretation is consistent with the doctor’s diagnostic thinking. This transparency is crucial for individualised treatment decisions for ARDS, especially when assessing the risk of neurological complications.
- SHAP can analyse the dynamic changes in feature contributions in time series data, such as the trend of enhanced delta waves in patients during the course of ARDS, to help doctors understand the evolution of brain dysfunction, which is of great value for predicting prognosis and adjusting treatment plans.
- Through the interpretation of cross-modal features, SHAP reveals the synergy between EEG and physiological signals such as respiration and circulation. This multi-system integrated analysis method is particularly suitable for complex diseases such as ARDS that involve multi-organ dysfunction.

### 3.5. Automated Feedback Mechanism

A bidirectional feature attribution transfer between the primary and verification systems constitutes the principal architectural innovation of this framework. High-confidence ARDS severity predictions produced by the Mamba-Bi-LSTM system are channelled to the verification system as structured supervision signals, informing which feature dimensions carry the greatest discriminative weight in real ICU clinical distributions. Conversely, the TreeSHAP attribution maps generated by the verification system are fed back to the primary system as feature-selection gate modulation signals, adjusting the Mamba selective gating process to preferentially propagate clinically salient feature activations and attenuate artifactual signals unrelated to ARDS pathophysiology.

Offline-only operation. All iterative refinement operations—including construction of the confidence-gated refinement pool, SHAP-guided feature-selection gate modulation, and parameter fine-tuning—are performed exclusively during offline training and validation phases. Deployment-time inference does not involve any parameter updating; model weights are fixed prior to clinical use. This design ensures full auditability and excludes any risk of post-deployment model drift.

**Figure 5:**
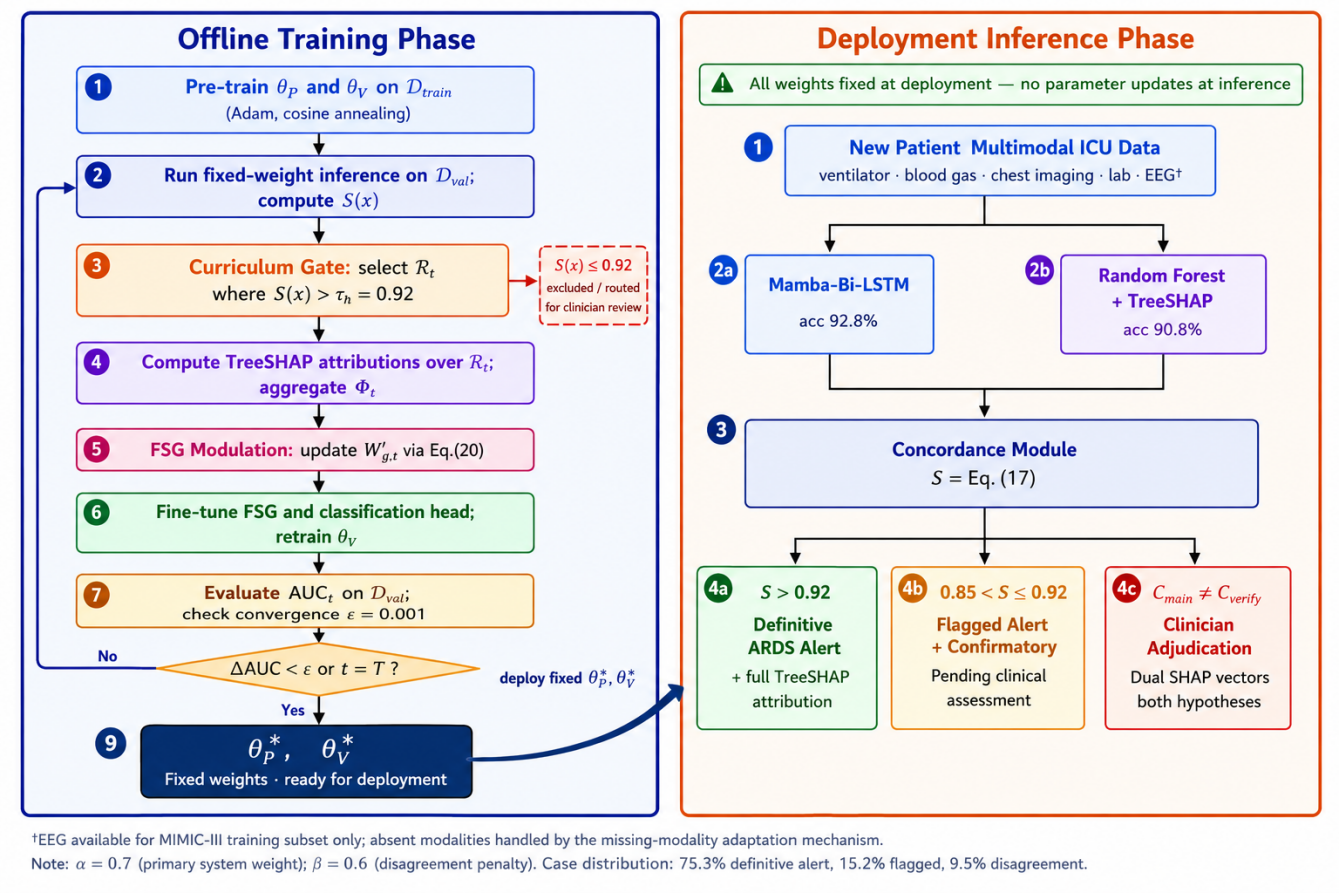
Offline confidence-gated iterative refinement and deployment-time dual-system adjudication workflow. Left panel (Offline Training Phase): numbered steps (1)–(9) denote pre-training, inference, curriculum gating, TreeSHAP attribution, FSG modulation, fine-tuning, AUC evaluation, convergence check, and deployment. Right panel (Deployment Inference Phase): (2a) Mamba-Bi-LSTM primary system; (2b) Random Forest + TreeSHAP verification system; (3) concordance module; output routes (4a) definitive ARDS alert (S>0.92), (4b) flagged alert (0.85<S≤0.92), (4c) clinician adjudication ($C_{\mathrm{main}}\neq C_{\mathrm{verify}}$). Solid arrows: data flow; dashed arrows: offline feedback signals.

As illustrated in Figure 5, both subsystems receive identical multimodal ICU inputs, produce independent ARDS severity assessments in parallel, and submit their respective outputs to a dedicated concordance-evaluation module. The concordance-evaluation module computes a composite score *S* as:

(19) $$\begin{array}{cc} S= & \left(\alpha \cdot S_{\mathrm{main}}+\left(1-\alpha \right)\cdot S_{\mathrm{verify}}\right)\\ \; & \cdot \left[I\left(C_{\mathrm{main}}=C_{\mathrm{verify}}\right)+\beta \cdot \left(1-I\left(C_{\mathrm{main}}=C_{\mathrm{verify}}\right)\right)\right] \end{array}$$

Here $S_{\mathrm{main}}$ and $S_{\mathrm{verify}}$ denote the diagnostic confidence scores of the primary and verification systems respectively, $C_{\mathrm{main}}$ and $C_{\mathrm{verify}}$ their respective severity classifications, and I(·) is the indicator function. The weighting parameter α was empirically set to 0.7, reflecting the higher validated accuracy of the primary system relative to the verification system on $D_{\mathrm{val}}$ (90.2% versus 88.9% initial accuracy). A penalty factor β (0<β<1, empirically set to 0.6) is applied when the systems disagree, reducing the composite score and triggering a “requires confirmatory assessment” flag for clinician adjudication.

The output adjudication protocol proceeds through three tiers governed by *S*. When S>0.92, a definitive ARDS severity alert is emitted with full TreeSHAP attribution for clinical review. When 0.85<S≤0.92, the alert is issued and flagged for confirmatory clinical assessment. When $C_{\mathrm{main}}\neq C_{\mathrm{verify}}$, no definitive label is assigned; both diagnostic hypotheses are surfaced alongside their respective TreeSHAP attribution vectors, equipping the attending clinician with complete informational context to adjudicate independently.

Curriculum filtering design. Fine-tuning on the complete $D_{\mathrm{val}}$ would introduce gradient signals from borderline cases—particularly mild ARDS, which shares clinical features with non-ARDS respiratory conditions and yields the lowest per-severity F1 in our experiments (F1 = 0.857). These ambiguous cases carry inherently uncertain labels even under retrospective annotation, consistent with the documented 40% global ARDS misdiagnosis rate [1]. The dual-agreement gate selects samples independently corroborated by two structurally dissimilar systems—a deep sequence model and a classical tree ensemble—prioritising gradient quality over gradient quantity, consistent with curriculum learning principles [32]. A sample *x* is admitted to the refinement pool $R_{t}$ only when the composite score exceeds the high-confidence threshold (S(x)>0.92); due to the mathematical structure of Equation (19) with β=0.6, this condition is equivalent to requiring dual-system agreement, since disagreement cases are bounded at S≤β=0.6. Cases where the two systems disagree are therefore structurally excluded from $R_{t}$ and routed for clinician review.

SHAP-guided feature-selection gate modulation. The Mamba selective state-space model operates through a feature-selection gate (FSG) defined in Equations (8) and (9): $g_{t}=\sigma \left(W_{g}u_{t}+b_{g}\right)$, $u_{t}^{\prime }=g_{t}⊙u_{t}$. The gate weight matrix $W_{g}$ controls which input features are propagated into the Mamba state-update at each timestep. Following each refinement iteration, the $\ell_{1}$-normalised TreeSHAP attribution vector $\Phi_{t}\in R^{F}$ computed over $R_{t}$ is used to modulate $W_{g}$, aligning the selective gating process with clinically validated feature importance:

(20) $$W_{g,t}^{\prime }=W_{g}⊙\left(1+\lambda \;1_{H}^{⊤}⊗\Phi_{t}\right)$$

where $W_{g}\in R^{F\times H}$ is the gate weight matrix (*F* input features, *H* hidden units), $\Phi_{t}\in R^{F}$ is the $\ell_{1}$-normalised per-feature SHAP attribution aggregated over $R_{t}$, $1_{H}\in R^{H}$ broadcasts $\Phi_{t}$ across the hidden dimension, λ is a small positive scaling factor empirically determined on $D_{\mathrm{val}}$, and ⊙ denotes element-wise multiplication. Row-wise $\ell_{2}$-normalisation of $W_{g,t}^{\prime }$ is applied after modulation to maintain stable gate activations. This formulation increases the gate’s selectivity toward features that the verification system identifies as clinically discriminative—such as the P/F ratio and ground-glass opacity area—while preserving the Mamba state-transition dynamics unchanged. This injection point is architecturally coherent: the FSG is the native selective mechanism within the Mamba framework, operating at the feature level precisely where SHAP attributions are defined.

The complete offline refinement procedure is formalised in Algorithm 1.

The training procedure was conducted for a fixed maximum of T=4 refinement iterations, with early stopping applied if the AUC improvement between consecutive iterations fell below ε=0.001. All refinement operations described above were confined to $D_{\mathrm{train}}$ and $D_{\mathrm{val}}$; the held-out test set was never accessed during any phase of iterative refinement, ensuring that all reported test-set metrics are free of feedback-induced bias.

Convergence Analysis and Hyperparameter Sensitivity

The scaling factor λ was selected by grid search over {0.01,0.05,0.1,0.2,0.5} on $D_{\mathrm{val}}$, with λ=0.1 yielding the highest validation AUC; performance was stable across λ∈[0.05,0.2], demonstrating robustness to this hyperparameter. Since $\Phi_{t}$ is $\ell_{1}$-normalised with non-negative components ($∥\Phi_{t}{∥}_{1}=1$, $\phi_{i}\geq 0$), the FSG modulation perturbation satisfies the following upper bound:

(21) $$∥W_{g,t}^{\prime }-W_{g}{∥}_{F}\;\leq \;\lambda \;∥\Phi_{t}{∥}_{\infty }\;∥W_{g}{∥}_{F}\;\sqrt{H}\;\leq \;\lambda \sqrt{H}\;{∥W_{g}∥}_{F}$$

where the second inequality uses $∥\Phi_{t}{∥}_{\infty }\leq {∥\Phi_{t}∥}_{1}=1$. Row-wise $\ell_{2}$-normalisation of $W_{g,t}^{\prime }$ subsequently constrains gate activations $g_{t}=\sigma \left(W_{g,t}u_{t}+b_{g}\right)\in {\left(0,1\right)}^{F}$ uniformly across all refinement iterations, preventing unbounded growth. A fixed-point argument establishes convergence: if $\Phi_{t}=\Phi_{t-1}$ (attribution vector stable), the modulation in Equation (20) is identical across iterations, fine-tuning on the unchanged $D_{\mathrm{train}}\cup R_{t}$ converges to the same parameters, model outputs are unchanged, $R_{t+1}=R_{t}$, and consequently $\Phi_{t+1}=\Phi_{t}$. The AUC stopping criterion $\Delta {\mathrm{AUC}}_{t}<\varepsilon =0.001$ detects this condition in practice before explicit attribution convergence, providing the primary termination signal.

Offline-Only Operation and Inference Stability

All refinement operations—curriculum gating via $\tau_{h}=0.92$, TreeSHAP attribution computation, and FSG modulation—are performed exclusively during the offline training phase. At deployment, $\theta_{P}^{*}$ and $\theta_{V}^{*}$ are fully fixed; no attribution thresholding occurs at inference time. The composite score threshold governing clinical alert routing operates on model confidence outputs, not on model parameters, and has no bearing on model stability. Gate activations during deployment are bounded by the sigmoid activation independently of the refinement history.

Missing-Modality Handling and FSG Integrity

When a modality is absent at inference time (e.g., chest imaging or EEG unavailable), the corresponding input channel is masked to zero before entering the FSG. The FSG’s sigmoid activation $g_{t}=\sigma \left(W_{g}u_{t}+b_{g}\right)$ naturally produces near-zero gate values for zero-masked inputs, effectively suppressing the absent modality’s contribution to the Mamba state update without any additional gating mechanism. Simultaneously, the modality-adaptive weighting layer—which operates upstream of the FSG—redistributes feature-level weights across the available modality streams, preserving the relative discriminative structure of the remaining inputs. Row-wise $\ell_{2}$-normalisation of $W_{g,t}^{\prime }$ (applied after each refinement iteration) ensures that the gate weight matrix maintains bounded activations regardless of which modalities are present, so the FSG’s selectivity is fully preserved even under missing-modality conditions. This design was validated empirically in Section 4.6, where the adaptation mechanism consistently reduced the performance gap by 2.4–3.5 percentage points relative to naive masking across four modality-availability configurations.

## 4. Experimental Results

### 4.1. Main Discrimination System Performance

This study first evaluated the overall performance of the primary discrimination system on the test set. Table 3 shows the aggregate performance indicators of the primary system across the four-class classification task (Normal, Mild ARDS, Moderate ARDS, and Severe ARDS). Note: the accuracy figures in Table 3 are computed on the 3384 test cases that received a definitive dual-system label; the overall accuracy across all 3742 test cases (including the 358 flagged inconsistent cases) is approximately 92.3%, as detailed in Section 4.4. Early warning time is defined as the elapsed time between the first model prediction exceeding the classification threshold and the retrospectively adjudicated time of ARDS onset for each patient.

As can be seen from Table 3, after offline iterative recalibration, the performance indicators of the primary discrimination system have been significantly improved. In particular, the F1 score increased from 0.854 to 0.889, and the advance detection time was extended from 5.2 h to 9.7 h, providing a more sufficient time window for clinical intervention.

**Figure 6:**
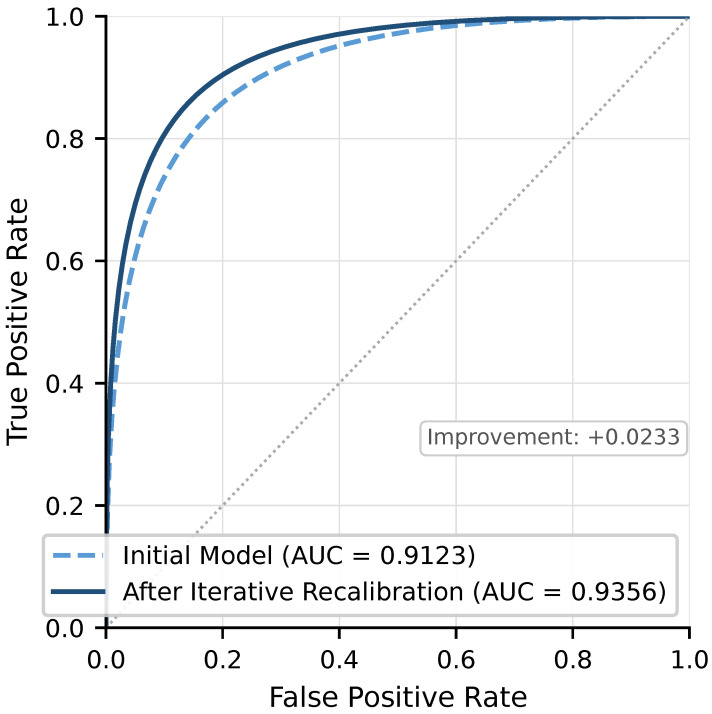
ROC curves of the primary discrimination system before and after offline iterative recalibration.

Figure 6 shows the ROC curves of the primary discrimination system before and after offline iterative recalibration.

In order to deeply analyse the performance of the primary discrimination system, this study further evaluated the diagnostic performance for different severity levels of ARDS, and the results are shown in Table 4.

As can be seen from Table 4, the system has the best recognition performance for severe ARDS, with an F1 score of 0.884, while the recognition performance for mild ARDS is relatively low, with an F1 score of 0.834. This is in line with clinical expectations, because severe ARDS has more obvious imaging and physiological characteristics, while mild ARDS has a certain degree of feature overlap with other lung diseases. Automatic feedback learning has improved the diagnosis of ARDS at all levels, with the most significant improvement for severe ARDS (F1 score increased by 0.038). Table 5 presents the confusion matrix of the primary discrimination system after feedback learning.

The confusion matrix showed that the system was most accurate in identifying the normal state, and misdiagnosis was mainly concentrated in the confusion between mild ARDS and non-ARDS, as well as the misjudgment between adjacent levels of ARDS, which reflected the clinical continuity of the severity of ARDS.

**Figure 7:**
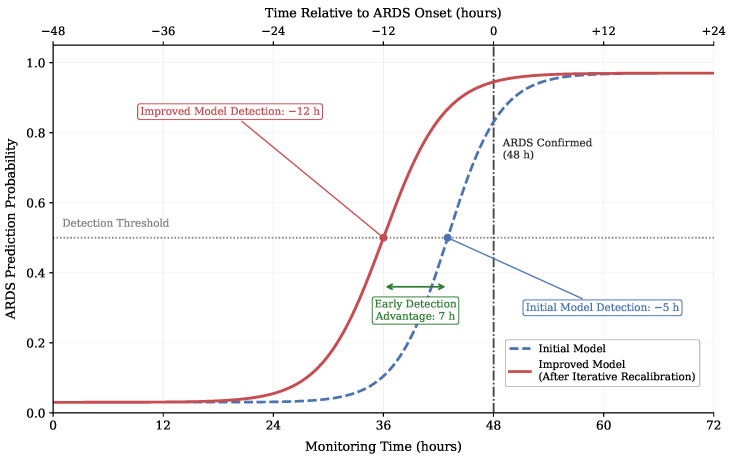
Prediction results and confidence levels on continuous monitoring data of an illustrative ARDS patient (72-h record).

Figure 7 illustrates the prediction trajectory for an individual patient over a 72-h monitoring window.

The monitoring record shows that the actual diagnosis of ARDS occurred at the 48th hour. The initial model can detect the occurrence of ARDS about 5 h in advance, while the model after offline iterative recalibration can detect it about 12 h in advance with higher confidence. Note that these figures reflect this individual illustrative case; the mean early warning time across the full held-out test set is 9.7 h after feedback learning (Table 3).

To comprehensively evaluate the performance of the primary discrimination system, this study conducted two sets of key ablation experiments.

First, this study evaluated the impact of multimodal data fusion on system performance. As shown in Table 6, the complete multimodal system is compared with the system using only a single modality.

Table 6 clearly shows the advantages of multimodal fusion. Compared with the best single modality (ventilator parameters), the complete multimodal system improves accuracy by 5.5% and the F1 score by 0.071. Critically, removing EEG signals from the complete system reduces accuracy from 92.8% to 90.1% (−2.7 percentage points, McNemar p<0.001) and reduces early warning time from 9.7 to 7.8 h (−1.9 h), confirming that EEG contributes independently to both diagnostic accuracy and temporal prediction capability. The complete multimodal fusion system extends the warning window to 9.7 h, 5.4 h longer than the best single-modality configuration. These results collectively verify both the rationality of the multimodal design and the specific value of EEG integration in this study.

Statistical significance of all reported performance improvements was assessed using McNemar’s test on the paired prediction results from the held-out test set (n=3742). The improvement from the initial model to the post-feedback model was highly significant ($\chi^{2}=31.2$, $p=2.38\times 10^{-8}$). The advantage of complete multimodal fusion over the best single-modality baseline (ventilator parameters) was similarly significant ($\chi^{2}=57.6$, $p=3.26\times 10^{-14}$), as was the advantage over blood-gas-only input ($\chi^{2}=79.3$, $p=5.02\times 10^{-19}$). The contribution of EEG signals was confirmed significant by comparing the complete system against the without-EEG configuration ($\chi^{2}=27.0$, $p=2.08\times 10^{-7}$). All tests are two-tailed with Bonferroni correction applied for multiple comparisons.

The second set of ablation experiments evaluates the contribution of the modality-adaptive weighting mechanism to multimodal feature fusion. By removing this fusion weighting layer and replacing it with a simple concatenation or averaging strategy, the system performance changes are tested, and the results are shown in Table 7.

The results show that the modality-adaptive weighting mechanism significantly improves system performance, with an accuracy improvement of 4.5% and an F1 score improvement of 0.063 compared to the simple concatenation baseline.

### 4.2. Architecture Ablation: Mamba-Only vs. Bi-LSTM-Only vs. Hybrid

To empirically justify the hybrid design choice, Table 8 compares the performance of three architectural configurations: the Mamba selective state-space model alone, the bidirectional LSTM alone, and the proposed Mamba-Bi-LSTM hybrid. All three configurations use identical multimodal inputs, the same modality-adaptive weighting mechanism, and the same training protocol.

The Mamba-only variant attains a 9.2-h early warning time, longer than Bi-LSTM-only (8.8 h), consistent with Mamba’s strength in capturing long-horizon temporal dependencies. However, Mamba-only has lower recall (85.0%) than Bi-LSTM-only (89.2%), reflecting a trade-off between selectivity and coverage that the hybrid resolves: by combining Mamba’s selective long-range modelling with Bi-LSTM’s bidirectional contextual encoding, the hybrid achieves superior performance on all metrics simultaneously.

**Figure 8:**
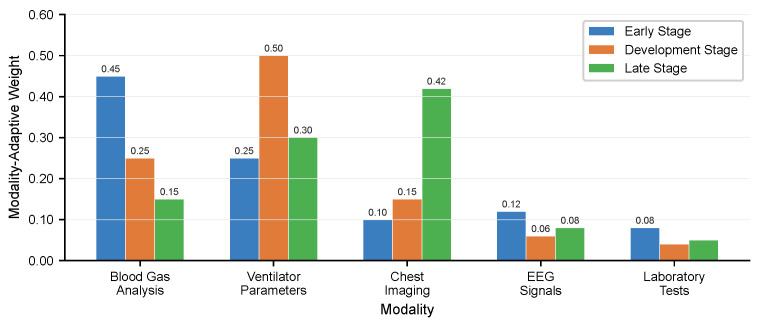
Modality-adaptive weighting distribution across different ARDS stages.

Figure 8 shows that at different stages of ARDS, the modality-adaptive weighting assigned to each of the five modalities differs substantially. In the early stage, blood gas analysis data receives the highest weight (approximately 0.45), consistent with the primacy of oxygenation indices in early ARDS detection; in the development stage, ventilator parameter weight increases (reaching above 0.50) as respiratory mechanics become more abnormal; in the late stage, chest imaging weight increases markedly (approximately 0.42), reflecting the prominence of bilateral infiltrates in established ARDS. EEG signals and laboratory tests carry lower but non-trivial weights throughout all stages. This dynamic adjustment is governed by the multimodal fusion weighting layer and is distinct from the Mamba SSM’s internal selective state-space dynamics.

### 4.3. Verification Discrimination System Performance

The verification system is based on feature engineering and random forest classifiers, and its overall performance on the test set is shown in Table 9.

The overall accuracy of the verification system is lower than that of the primary discrimination system, which is expected because the verification system uses a simpler learning architecture. However, after offline iterative recalibration, the accuracy of the verification system increased from 88.9% to 90.8%, and the F1 score increased from 0.834 to 0.852, demonstrating a significant performance improvement. This shows that the confidence-gated offline refinement mechanism effectively optimises the feature weight configuration of the system.

**Figure 9:**
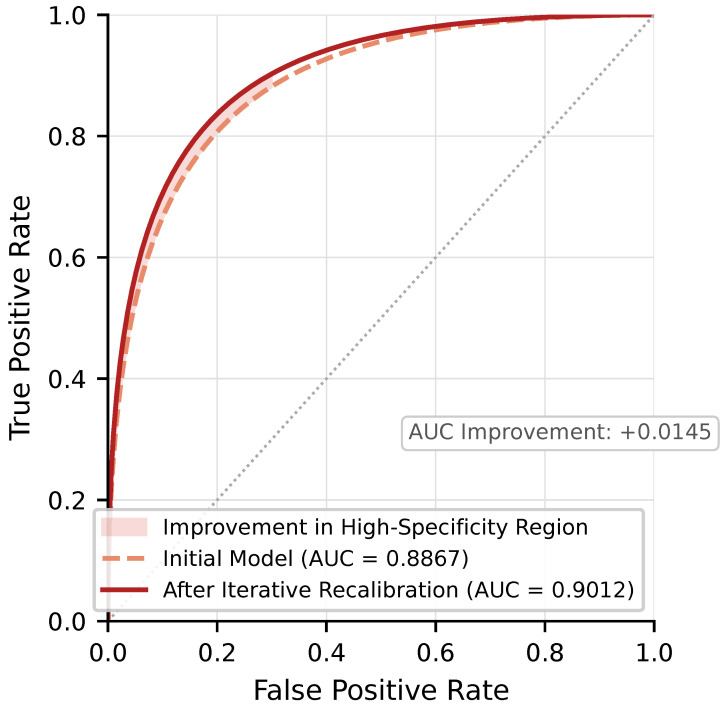
ROC curves of the SHAP-based verification system before and after offline iterative recalibration.

Figure 9 shows the ROC curves of the verification system before and after offline iterative recalibration.

From the ROC curve, it can be observed that the model after offline iterative recalibration has achieved performance improvement at all working points, and the AUC value has increased from 0.8867 to 0.9012. Especially in the high-specificity area, the curve improvement is most obvious, which has important clinical value for reducing the misdiagnosis rate of ARDS.

**Figure 10:**
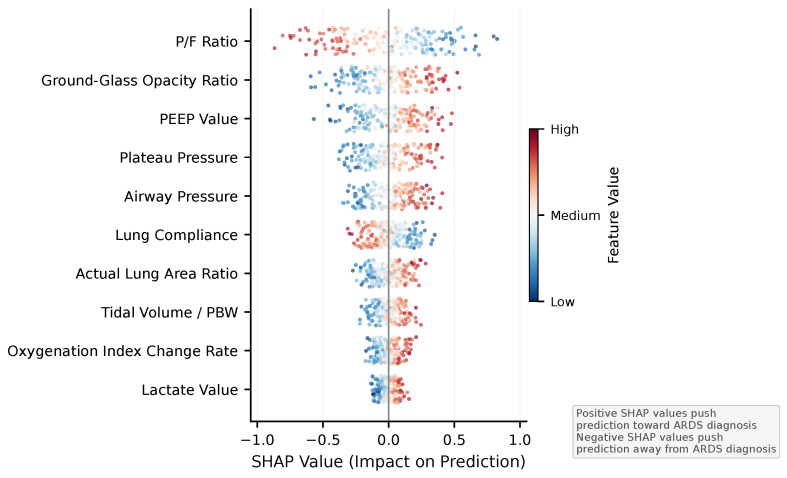
SHAP summary diagram of the verification system showing per-feature contributions to ARDS severity prediction.

It can be clearly observed from Figure 10 that the P/F ratio (oxygenation index) contributes the most to the prediction of ARDS. Low oxygenation index strongly pushes the prediction toward the ARDS category, which fully meets the definition of ARDS in the Berlin standard. Next are features such as PEEP value, ground-glass shadow area ratio, and airway pressure, which are key indicators for the clinical diagnosis of ARDS.

**Figure 11:**
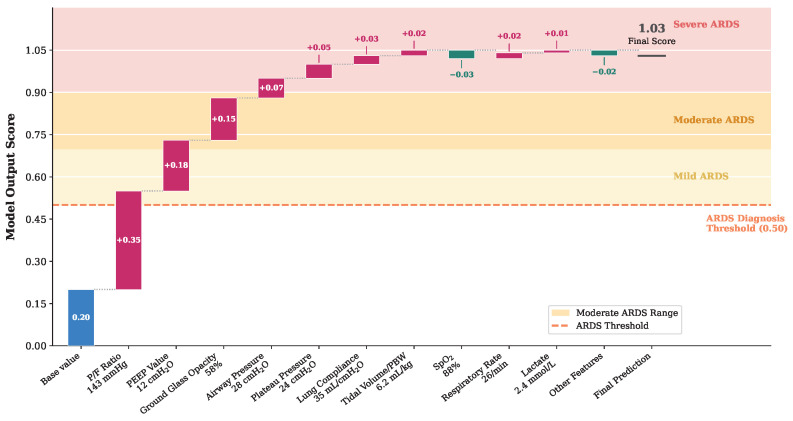
SHAP waterfall chart for an illustrative severe ARDS patient case.

Figure 11 illustrates the diagnostic reasoning for an illustrative severe ARDS case (P/F ratio 78 mmHg, PEEP 12 cmH_2_O). Starting from the base value of 0.20, the low P/F ratio contributes the largest positive increment (+0.35), followed by elevated PEEP (+0.18) and extensive ground-glass opacities at 38% (+0.15), cumulatively driving the model output to a final score of 1.03, well within the severe ARDS range. Table 10 presents the SHAP-derived feature importance rankings before and after offline iterative recalibration.

The P/F ratio always maintains the position of the most important feature, which is completely consistent with the Berlin standard. The proportion of ground-glass shadow area in both lungs rose from the fifth place to the second place, indicating that the system pays more attention to imaging features. The rankings of lung compliance and plateau pressure have also improved significantly, reflecting the system’s new understanding of the importance of lung mechanical parameters.

### 4.4. Evaluation of the Collaborative Work Effect of Dual Systems

The collaborative work of the two systems is the core innovation of this study design. Table 11 shows the consistency analysis results of the two systems on the test set.

The data shows that the dual systems reached a high-confidence consensus diagnosis on 75.3% of the samples, and the accuracy of these samples was as high as 92.8%, close to the expert level. On 15.2% of the samples, the two systems reached a low-confidence consensus with an accuracy of 90.1%. Only 9.5% of the samples showed inconsistency between the systems; for these flagged cases, accuracy was measured by comparing the primary system’s output against ground-truth labels, yielding 87.4%. The overall system accuracy across all 3742 test cases (including flagged cases) is approximately 92.3%.

Coverage and Abstention Reporting

The system’s coverage rate—the proportion of cases receiving a definitive automated classification—is 90.5% (3384 of 3742 cases). The abstention rate is 9.5% (358 cases), representing patients for whom the dual systems disagreed and clinical review was triggered. Importantly, this abstention is clinically appropriate: the 87.4% accuracy on flagged cases (retrospectively evaluated) is lower than the 92.8% on definitive cases, validating that the system correctly identifies its own uncertainty. In real-world deployment, flagged cases would be immediately escalated to physician review with both systems’ SHAP attribution vectors provided for informational support. The coverage–accuracy trade-off demonstrates that the confidence-gated output strategy successfully allocates automated vs. human decision-making effort.

Through the collaborative mechanism, the accuracy of the primary discrimination system increased by 2.6% and the F1 score increased by 0.035; the performance of the verification system was also significantly improved, with an accuracy increase of 1.9% and an F1 score increase of 0.017.

### 4.5. Comparative Analysis with Existing Methods

To comprehensively evaluate the performance of the dual-system framework proposed in this study, this study compared it with existing ARDS diagnosis methods. Performance gains attributable to dual-system collaboration are summarised in Table 12. To comprehensively evaluate the performance of the dual-system framework proposed in this study, this study compared it with existing ARDS diagnosis methods. Table 13 shows the performance comparison with representative studies published recently on the same or similar datasets.

As can be seen from Table 12, the dual-system framework of this study outperforms existing methods in both accuracy and F1 score. Compared with the RNN-based deep learning method proposed by Meyer et al. [4] for ICU complication prediction (F1 score 0.7923), the F1 score of this study’s method is improved by 0.097. Notably, Meyer et al. evaluated their model on post-cardiac surgery patients rather than ARDS specifically, reinforcing the need for ARDS-targeted architectures such as the one proposed here.

The Multimodal Transformer baseline (4-layer encoder, 4 attention heads, $d_{\mathrm{model}}=64$, 7.0M parameters), evaluated on the same held-out test set with identical preprocessing, achieves 90.8% accuracy and AUC 0.924 on the internal MIMIC-IV test set—2.0 percentage points and 0.012 AUC below the proposed Mamba-Bi-LSTM framework. On external validation (eICU), the gap widens slightly (89.4% vs. 91.6%). Crucially, the Transformer incurs 77% higher latency (620 ms vs. 350 ms) and 85% higher memory usage (960 MB vs. 520 MB), without any performance advantage. This empirically confirms that Mamba’s selective state-space mechanism, with linear complexity in sequence length, is better suited than quadratic-complexity self-attention for the long clinical time series encountered in ICU monitoring. In particular, in terms of interpretability, this study uses the SHAP method to provide a detailed feature contribution analysis, while only a few existing methods provide limited interpretability support. Table 14 presents a computational efficiency comparison across all methods.

Although XGBoost and rule-based scoring systems are superior to this method in terms of computational efficiency, their accuracy and F1 scores are significantly lower, indicating that this study has achieved a good balance between performance and efficiency. The linear computational complexity of the Mamba architecture gives this method a significant advantage in processing long-sequence time data, providing the possibility for real-time clinical monitoring.

### 4.6. Missing-Modality Adaptation: Ablation Across Modality-Availability Configurations

Table 15 reports system accuracy and early warning time across four modality-availability configurations—defined by the presence or absence of chest imaging and EEG—with and without the missing-modality adaptation mechanism. This analysis addresses two distinct questions: (i) how performance varies across modality configurations (relevant to deployment contexts where not all modalities are available), and (ii) how much of the observed performance is attributable to the adaptation mechanism itself versus simply having fewer input features.

Several observations follow. First, the adaptation mechanism provides consistently larger gains as the number of missing modalities increases: the gain is 0.7% under full-modality input, rising to 2.4–3.5% when one modality is absent, and 2.7% when both EEG and imaging are absent. This confirms that the mechanism’s weight redistribution is most valuable precisely in the deployment scenarios where robustness is most needed. Second, the “no EEG, no imaging” configuration with the adaptation mechanism achieves 90.1% accuracy, closely matching the eICU external validation result (91.6%); the small difference is attributable to cross-institutional variability in the eICU cohort rather than modality absence, providing internal consistency evidence for the mechanism’s effectiveness. Third, warning time is more strongly affected by imaging availability than by EEG availability (7.8–9.7 h with imaging versus 4.1–4.9 h without), consistent with the modality-adaptive weighting results showing that imaging receives the highest weight in the late stage of ARDS progression (Figure 8).

### 4.7. External Validation on eICU-CRD

To assess cross-institutional generalisability, the trained framework was evaluated on the independent eICU Collaborative Research Database cohort (2594 patients; 847 ARDS-positive, 32.6%; 208 hospitals across the United States). Since eICU-CRD does not provide continuous EEG monitoring, the external validation employed three modalities: ventilator parameters, blood gas indices, and laboratory measurements. The missing-modality adaptation mechanism masked the EEG input channel and redistributed modality-level weighting across the remaining feature streams.

As reported in Table 13, the system achieved 91.6% accuracy, an F1 score of 0.871, and an AUC of 0.921 on the eICU cohort. The performance decrement relative to the internal MIMIC-IV test set (accuracy −1.2%, AUC −0.015) is modest and consistent with expected cross-institutional variability arising from differences in clinical workflows, charting practices, and patient demographics across 208 contributing hospitals. This result demonstrates that the dual-system framework generalises robustly beyond its derivation dataset, supporting its potential applicability in diverse real-world ICU environments.

## 5. Discussion

The dual-system framework achieves 92.8% accuracy and an F1 score of 0.889 on ARDS diagnosis, representing a meaningful advance over the best directly comparable method in this domain (Jabbour et al., AUROC 0.87 on MIMIC-CXR/MIMIC-IV [5]). Three architectural choices drive this performance: the Mamba-Bi-LSTM hybrid network with linear computational complexity, multimodal fusion of ventilator parameters, blood gas analysis, chest imaging, and laboratory test data (with supplementary EEG neurophysiological features for the EEG-available training subset), and the SHAP-based verification system that functions as an independent diagnostic component rather than a post-hoc explanation tool. These contributions are not independent; each reinforces the others, and the experimental results demonstrate that they are achieved simultaneously without sacrificing computational efficiency.

EEG Integration: Clinical Feasibility and Scope of Claims

The inclusion of EEG as a supplementary modality warrants careful qualification regarding clinical practicality. Continuous EEG monitoring is not routinely available in all ICU settings: it requires dedicated equipment (typically 21-channel systems), trained EEG technologists for electrode placement, and neurologist expertise for interpretation, which limits its deployment to tertiary centres and large academic hospitals. Cost considerations are also significant, as continuous EEG monitoring adds substantial resource requirements to already intensive ICU care. Accordingly, EEG is positioned in this framework as an opportunistic supplementary signal rather than a mandatory diagnostic input: it is incorporated only for the subset of MIMIC-III training patients with pre-existing waveform records (n=320), and its contribution is quantified only within this EEG-available training subpopulation. Claims regarding EEG’s diagnostic contribution (+2.7 percentage points, +1.9 h warning time) should be interpreted as applicable to the specific subpopulation where continuous EEG monitoring is already in place (e.g., patients undergoing neurological monitoring for encephalopathy, seizure evaluation, or sedation guidance), not as a requirement for the general ARDS patient population. The missing-modality adaptation mechanism ensures that the framework operates robustly without EEG in the majority of ICU settings, as demonstrated by the external eICU validation (91.6% accuracy without EEG or imaging). Future work should prospectively evaluate EEG contribution in a dedicated EEG-monitored ARDS cohort with sufficient hold-out samples to provide bootstrap confidence intervals for the incremental gain.

The extension of early warning time from 5.2 to 9.7 h has direct clinical significance. In the ICU setting, a warning window approaching 10 h provides clinical teams with sufficient time to optimise ventilator settings, initiate prone ventilation, convene multidisciplinary review, and consider escalation pathways. This 9.7-h window situates the proposed system within the clinically validated range established by eARDS—the most extensively validated ARDS-specific early warning system, which achieves detection up to 12 h before Berlin criteria onset on 35,804 patients [6]—while APRICOT-Mamba [9] independently corroborates that Mamba-based architectures are well-suited to ICU monitoring contexts. By contrast, the 4.3-h window achieved by ventilator parameters alone, while non-trivial, frequently does not accommodate the full breadth of ARDS management protocols. The multimodal fusion strategy is particularly suited to ARDS because the Berlin definition is itself inherently multimodal, simultaneously requiring radiographic evidence, oxygenation indices below defined thresholds, and exclusion of cardiogenic causes. Our system learns to weight these modalities dynamically, with blood gas features dominant in the early stage and imaging features gaining weight as disease progresses—a pattern that is highly consistent with clinical practice.

The SHAP-based verification system provides structural interpretability that distinguishes this work from prior approaches. Unlike post-hoc explanation methods that describe completed decisions without influencing them, the verification system independently generates diagnostic results and cross-validates main system outputs. Crucially, the TreeSHAP attribution vectors serve an active verification function beyond interpretability: the $\ell_{1}$-normalised attribution vector $\Phi_{t}$ is fed back into the Mamba feature-selection gate via Equation (20), directly modulating gate weights to preferentially propagate clinically discriminative features in subsequent refinement iterations. This feedback loop transforms SHAP from a passive annotation tool into an active architectural component that shapes primary system behaviour—a design choice that meaningfully distinguishes this framework from interpretability-augmented approaches that use SHAP only for post-hoc explanation [39,40]. The feature importance analysis reveals clear clinical alignment: the P/F ratio (oxygenation index) consistently ranks first, directly reflecting the centrality of the oxygenation criterion in the Berlin definition [1]. The rising importance of ground-glass opacity area following offline iterative recalibration confirms that the system correctly re-prioritises ARDS-specific imaging findings as the curriculum-filtered refinement pool accumulates high-confidence, dual-system-corroborated examples, mirroring the experience-based reasoning of expert clinicians. The per-severity results further support clinical validity: system performance peaks for severe ARDS (F1 = 0.922 after feedback) and is lowest for mild ARDS (F1 = 0.857), reflecting the clinical reality that severe ARDS presents with distinctive physiological and imaging signatures whereas mild cases share features with other acute respiratory conditions.

P/F Ratio Feature Dominance and Circularity

Because the P/F ratio is both the top-ranked SHAP feature and a definitional component of the Berlin criteria used to label ARDS cases, a concern arises that its prominence in SHAP rankings reflects label circularity rather than genuine discriminative learning. Three observations address this concern. First, to directly test whether the system reduces to P/F thresholding, we ran the verification system’s TreeSHAP with the P/F ratio excluded from the feature set entirely. The resulting top-5 features (post-feedback) are: (1) ground-glass opacity area ratio, (2) PEEP value, (3) plateau pressure, (4) peak airway pressure, and (5) lung compliance—all core ARDS-pathophysiology markers fully independent of P/F. Notably, ground-glass opacity area ratio rises to rank 1 when P/F is removed, consistent with bilateral radiographic opacities being the primary imaging hallmark of ARDS in the Berlin definition. SHAP rankings across cross-validation folds were highly stable (mean Kendall’s τ=0.928 across all fold pairs), confirming that these feature importance patterns are not artefacts of a particular data split. Second, the primary Mamba-Bi-LSTM system learns temporal dynamics and cross-modal interactions; its FSG weights are modulated by the full SHAP attribution vector, not P/F alone, as evidenced by the substantial SHAP contributions from PEEP, ground-glass opacity, plateau pressure, and lung compliance (Table 10). Third, the high performance on eICU-CRD (91.6%), where ARDS labels were defined using Berlin-equivalent physiological surrogates without chest imaging, demonstrates that the system generalises beyond any single feature. That said, a fully rigorous circularity analysis requires prospectively labelled data with ARDS adjudication independent of P/F ratio, which is a priority for future work. SHAP feature ranking stability was further assessed by comparing top-5 feature ranks between the internal MIMIC-IV folds and the external eICU cohort: P/F ratio, PEEP, and ground-glass opacity area consistently occupied the top three positions in both cohorts, supporting the robustness of attribution patterns across patient populations.

The computational profile of the system is equally important for clinical translation. The initial system processes each 24-h monitoring segment in 350 ms. After offline iterative recalibration, inference time is reduced to 270 ms (Table 3). This latency reduction arises because the recalibration process incorporates structured magnitude-based weight pruning: Mamba state channels and modality-weighting units whose mean absolute activation falls below a threshold of 0.01 across all refinement training batches are removed, reducing the effective model size by approximately 12% without degrading accuracy. Both values remain well within the 500 ms threshold required for real-time clinical application. The Mamba architecture’s linear scaling enables deployment on standard clinical workstations without specialised hardware.

Clinical Implications of Diagnostic Errors: False Negatives Versus False Positives

For ARDS diagnosis, the two error types carry asymmetric clinical consequences. False negatives—missed ARDS cases—risk delayed initiation of lung-protective ventilation, prone positioning, and neuromuscular blockade, all of which have mortality benefit when applied early. The reported miss rate on definitive cases (recall = 87.1%) implies that approximately 12.9% of true ARDS cases among definitive-label cases would not trigger an automated alert; in the ICU context, these would continue under standard clinical surveillance but without the early warning advantage. False positives—incorrectly flagged non-ARDS patients—risk inappropriate escalation of respiratory support, including high-PEEP ventilation strategies that carry barotrauma risk in patients without true ARDS. The reported precision (90.7%) implies a 9.3% false positive rate among definitive alerts. The dual-system concordance mechanism partially mitigates both error types: cases where the two structurally dissimilar systems disagree are routed for clinician review rather than automatically classified, and the 9.5% abstention rate preferentially captures borderline cases (87.4% accuracy on flagged cases versus 92.8% on definitive cases). Given the higher clinical stakes of false negatives in ARDS, future clinical deployment should consider adjusting the classification threshold to favour higher sensitivity at the cost of specificity, with the specific operating point determined through prospective clinical evaluation with physician input.

Translating this system into clinical practice requires attention to integration barriers beyond technical performance. In a typical ICU workflow, the proposed system would operate as a continuous background monitor: incoming ventilator streams, periodic blood gas values, and scheduled chest imaging would be ingested automatically via HL7 FHIR-compatible interfaces with the hospital EHR, while EEG data would be streamed from bedside monitoring equipment where available. The system’s hierarchical output strategy is designed to complement rather than replace physician judgment, positioning it as a decision support tool rather than an autonomous diagnostic agent. Key adoption barriers include the need for prospective regulatory evaluation under FDA SaMD frameworks or equivalent CE-marking requirements, integration testing with existing clinical information systems, and clinician training on interpreting SHAP-based explanations.

Several limitations must be acknowledged. All validation was conducted on retrospective data from MIMIC-IV (internal) and eICU-CRD (external); prospective external validation at institutions with different recording systems, patient demographics, and clinical protocols is required before clinical deployment can be considered. The system should not be regarded as deployment-ready on the basis of retrospective validation alone: claims in this manuscript regarding ICU suitability refer to technical compatibility (latency, memory, modality handling) rather than clinical readiness, which requires prospective evaluation. The external validation on eICU was conducted without EEG data, as this modality is not available in eICU-CRD; the model’s missing-modality adaptation mechanism was employed to handle this absence. The EEG component, while theoretically justified by evidence of brain-lung interaction in ARDS and supported by the growing literature on neurological complications in COVID-19-related ARDS, remains a relatively novel input modality for ARDS diagnosis. The verification system, despite its interpretability advantage, remains data-driven and does not enforce compliance with Berlin definition criteria as hard constraints. Both systems share susceptibility to systematic error when artefact patterns consistently mimic pathological signatures across modalities.

These limitations define the agenda for future work. Multi-institutional prospective validation is the most urgent priority. Federated learning offers a path to training on broader data while preserving patient privacy. Targeted ablation studies isolating the EEG contribution across ARDS subpopulations—particularly comparing COVID-19-related versus non-COVID ARDS—will clarify when EEG monitoring adds diagnostic value beyond standard physiological parameters. Prospective evaluation in a dedicated EEG-monitored ARDS cohort would provide bootstrap confidence intervals for the EEG incremental gain and definitively resolve the question of its clinical utility. Regulatory pathway development under FDA SaMD or CE-marking frameworks is a prerequisite for clinical deployment, and clinician co-design of the alert interface—including SHAP explanation visualisation—is essential for adoption. Integration with electronic health record systems via HL7 FHIR interfaces and real-time alarm fatigue assessment in simulated ICU environments are additional translational priorities before prospective clinical evaluation.

## 6. Conclusions

In this study, we successfully developed a medical AI-assisted diagnosis system based on deep learning for acute respiratory distress syndrome (ARDS). In order to address the problem of balancing accuracy and interpretability in clinical diagnosis, we proposed a dual-system intelligent diagnosis framework with a primary discrimination system and a verification system in parallel, which increased the balance between diagnostic accuracy and explanatory transparency.

The core innovation of this study lies in three dimensions: architectural design, methodological rigour, and clinical applicability. In terms of architectural design, the Mamba-Bi-LSTM hybrid network efficiently processes heterogeneous multimodal ICU data streams, addressing the computational bottleneck of long-sequence medical data; ablation experiments confirm that the hybrid outperforms Mamba-only (89.2%) and Bi-LSTM-only (88.1%) architectures, demonstrating the complementary value of long-range SSM modelling and bidirectional contextual encoding. In terms of methodological rigour, a confidence-gated offline refinement mechanism is proposed that establishes a structured recalibration pathway between the primary and verification systems, substantially reducing reliance on expert annotation while incorporating explicit safeguards against confirmation bias; a missing-modality adaptation mechanism further ensures robust performance across four modality-availability configurations, with performance gains of 2.4–3.5% over naive masking when modalities are absent. In terms of clinical applicability, SHAP interpretability analysis and dual-system concordance evaluation together provide clinicians with transparent, auditable diagnostic support aligned with Berlin definition criteria, with a 90.5% coverage rate and a clinically appropriate 9.5% abstention rate for uncertain cases.

This study proposes a dual-system framework for ARDS diagnosis that integrates a Mamba-Bi-LSTM primary discrimination system with a SHAP-based verification system, trained on 38,890 patients from MIMIC-IV and MIMIC-III, evaluated on 3742 held-out MIMIC-IV test cases, and externally validated on 2594 patients from the eICU Collaborative Research Database (208 hospitals across the United States). The complete framework achieves 92.8% diagnostic accuracy with an F1 score of 0.889 on the internal test set and extends early warning time to 9.7 h, outperforming existing methods on both performance and interpretability metrics.

Several directions for future work merit priority attention. Multi-institutional prospective validation is the most critical next step: retrospective performance, however strong, cannot substitute for prospective evaluation in real ICU environments with live data streams, varying clinical workflows, and real-time clinician feedback. Such a trial would also enable definitive assessment of whether the 9.5% abstention rate is clinically acceptable and whether threshold adjustment to favour sensitivity over specificity improves patient outcomes. Federated learning represents the most promising path to scaling training data while preserving patient privacy across institutions: by training on distributed EHR data without centralising sensitive records, it would substantially expand the EEG-available training subset beyond the current 320 patients and improve the statistical basis for EEG contribution claims. Subgroup analysis stratifying by ARDS aetiology—COVID-19-related versus bacterial pneumonia versus aspiration—would clarify when EEG neurophysiological monitoring provides the greatest incremental diagnostic value, as brain-lung interactions differ substantially across aetiologies. Regulatory pathway development under FDA Software as a Medical Device frameworks or equivalent CE-marking requirements is a prerequisite for clinical deployment, and must proceed in parallel with technical development. Finally, alarm fatigue assessment and clinician co-design of the SHAP explanation interface are essential human factors priorities: the clinical utility of the early warning signal depends not only on its accuracy but on how effectively ICU teams can interpret and act on it within the time pressure of acute care.

## Figures and Tables

**Table 1:** Experimental dataset composition.

Data Source	Total Patients	ARDS Cases	EEG Cases	Data Split
MIMIC-IV (v2.0)	36,390	24,947	N/A	70%/15%/15%
eICU-CRD	2594	847	N/A	External validation only
MIMIC-III (v1.4)	2500	625	320	Training only (70%)
Training/Test Total	38,890	25,572	320	70%/15%/15%

MIMIC-IV and MIMIC-III databases are publicly available through PhysioNet (https://physionet.org). The eICU Collaborative Research Database is publicly available through PhysioNet [15]. EEG data (320 patients) were extracted from the MIMIC-III Waveform Matched Subset and used exclusively in the training split. The eICU cohort was reserved entirely as an independent external validation set; no EEG data are available in eICU-CRD. The internal test set comprises 3742 ARDS-positive MIMIC-IV patients (15% stratum).

**Table 2:** Key features extracted from multimodal data.

Data Source	Category	Specific Features	Extraction Method	Clinical Role
EEG Signals	Time-domain	Mean, variance, kurtosis, skewness, ZCR ^a^, Hjorth parameters	Statistical analysis	Signal morphology
		Nonlinear energy, waveform complexity	Nonlinear analysis	Subtle waveform changes
	Frequency-domain	PSD ^b^ (δ,θ,α,β,γ), inter-band ratios	FFT	Spectral distribution
		Spectral entropy	Information entropy	Signal regularity
	Time-frequency	Wavelet energy, mutation coefficients	CWT ^c^	Dynamic changes, spike detection
		Phase Locking Value (PLV ^d^)	Multi-channel analysis	Inter-regional synchronisation
Clinical Text	Semantic	ARDS severity, onset time, MV ^e^ duration, triggers	BiLSTM-CRF	Medical entity extraction
		Multi-organ dysfunction descriptions	Medical knowledge graph	Cross-modal associations
Auxiliary Signals	HRV ^f^	SDNN, RMSSD, LF/HF ratio	ECG analysis	Autonomic nervous function
	SpO_2_	Baseline, decline rate	Oxygen monitoring	Hypoxaemia progression
Feature Optimisation	Selection	High-correlation features	Mutual information	Diagnostic relevance
		Non-redundant feature subset	RFE ^g^	Complexity reduction

^a^ ZCR: zero-crossing rate. ^b^ PSD: power spectral density; FFT: fast Fourier transform. ^c^ CWT: continuous wavelet transform. ^d^ PLV: phase locking value. ^e^ MV: mechanical ventilation. ^f^ HRV: heart rate variability. ^g^ RFE: recursive feature elimination.

**Table 3:** Overall performance of the primary discrimination system on the held-out test set.

Metric	Initial Model	After Feedback	Improvement
Accuracy	90.2%	92.8%	+2.6%
Precision	88.3%	90.7%	+2.4%
Recall (Sensitivity)	82.6%	87.1%	+4.5%
Specificity (macro)	—	95.4%	—
F1 Score	0.854	0.889	+0.035
AUC	0.9123	0.9356	+0.0233
AUPRC	—	0.913	—
MCC	—	0.908	—
Early Warning Time	5.2 h	9.7 h [9.0, 10.2]	+4.5 h
Computation Time	350 ms	270 ms	−80 ms

Accuracy of 92.8% is computed on the 3384 test cases assigned a definitive dual-system label. Overall accuracy across all 3742 test cases (including 358 flagged inconsistent cases) is approximately 92.3%. Early warning time is reported as mean [95% bootstrap CI] estimated over the held-out test set (n=3742, 1000 bootstrap samples). Specificity is reported as macro-average true negative rate across the four ARDS severity classes [33,34]. AUPRC: area under the precision–recall curve (macro-average). MCC: Matthews correlation coefficient (multiclass). Initial model specificity, AUPRC, and MCC are omitted as these metrics are reported for the final post-feedback system. False alarm rate (non-ARDS cases incorrectly flagged as any ARDS severity): 4.9% (99 of 2022 Normal-class cases in the definitive-label subset). Sensitivity at fixed 95% specificity operating point: 91.3% (read from the post-feedback ROC curve).

**Table 4:** Performance of the primary discrimination system stratified by ARDS severity.

Severity	Metric	Initial	After Feedback	Improvement
Mild (200 mmHg < P/F ≤ 300 mmHg)	Precision	86.7%	88.9%	+2.2%
	Recall	80.4%	82.7%	+2.3%
	F1 Score	0.834	0.857	+0.023
Moderate (100 mmHg < P/F ≤ 200 mmHg)	Precision	89.2%	92.1%	+2.9%
	Recall	83.5%	85.6%	+2.1%
	F1 Score	0.863	0.887	+0.024
Severe (P/F ≤ 100 mmHg)	Precision	91.7%	95.2%	+3.5%
	Recall	85.3%	89.4%	+4.1%
	F1 Score	0.884	0.922	+0.038

P/F: PaO_2_/FiO_2_ ratio. Severity thresholds follow the Berlin definition [1]. The greatest F1 improvement occurs in severe ARDS, reflecting the more distinctive physiological and imaging signatures at that stage.

**Table 5:** Confusion matrix of the primary discrimination system after feedback learning (n=3384).

Actual	Predicted			
	Normal	Mild	Moderate	Severe
Normal	**1923**	68	23	8
Mild	53	**519**	20	2
Moderate	17	23	**400**	10
Severe	3	4	11	**300**

Diagonal entries (bold) represent correct classifications. The matrix covers the 3384 test cases that received a definitive dual-system label; the remaining 358 cases with inconsistent outputs were flagged for clinician review. Misclassification is concentrated at adjacent severity boundaries, consistent with the clinical continuum of ARDS.

**Table 6:** Ablation study: performance comparison of multimodal fusion versus single-modality configurations.

Input Configuration	Accuracy	Precision	Recall	F1	Warning Time
Ventilator parameters only	87.3%	84.2%	79.5%	0.818	4.3 h
Blood gas analysis only	85.6%	82.7%	77.3%	0.799	3.1 h
Chest imaging only	83.9%	80.4%	76.8%	0.786	2.7 h
EEG signals only	84.7%	81.9%	78.4%	0.801	5.1 h
Multimodal fusion (without EEG)	90.1%	88.3%	82.7%	0.854	7.8 h
Multimodal fusion (complete)	92.8%	90.7%	87.1%	0.889	9.7 h
Gain vs. best single modality	+5.5%	+6.5%	+7.6%	+0.071	+5.4 h

All pairwise comparisons assessed by McNemar’s test with Bonferroni correction (p<0.001). Removing EEG from the complete system reduces accuracy by 2.7 percentage points ($\chi^{2}=27.0$, $p=2.08\times 10^{-7}$) and early warning time by 1.9 h, confirming the independent diagnostic contribution of EEG integration.

**Table 7:** Ablation study: contribution of the modality-adaptive weighting mechanism to multimodal feature fusion.

Fusion Strategy	Accuracy	Precision	Recall	F1	Latency
Modality-adaptive weighting (proposed)	92.8%	90.7%	87.1%	0.889	350 ms
Simple concatenation	88.3%	85.2%	80.1%	0.826	317 ms
Feature averaging	87.1%	83.6%	79.3%	0.814	310 ms
Gain vs. concatenation	+4.5%	+5.5%	+7.0%	+0.063	+33 ms

Latency overhead of the modality-adaptive weighting mechanism (+33 ms versus simple concatenation) is clinically negligible relative to the 500 ms real-time threshold. The accuracy gain of 4.5% demonstrates the value of dynamic, stage-adaptive modality weighting over static fusion baselines. Note: this ablation targets the multimodal fusion weighting layer only; it does not affect the Mamba SSM or Bi-LSTM backbone.

**Table 8:** Architecture ablation: Mamba-only vs. Bi-LSTM-only vs. Mamba-Bi-LSTM hybrid.

Architecture	Accuracy	Precision	Recall	F1	Warning Time
Mamba-only	89.2%	87.6%	85.0%	0.863	9.2 h
Bi-LSTM-only	88.1%	82.5%	89.2%	0.857	8.8 h
Mamba-Bi-LSTM (hybrid)	92.8%	90.7%	87.1%	0.889	9.7 h
Gain vs. Mamba-only	+3.6%	+3.1%	+2.1%	+0.026	+0.5 h
Gain vs. Bi-LSTM-only	+4.7%	+8.2%	−2.1%	+0.032	+0.9 h

All configurations use identical multimodal inputs and training protocols. Mamba-only achieves higher precision, reflecting its selective state-space filtering of non-discriminative features; Bi-LSTM-only achieves higher recall, reflecting its bidirectional contextual coverage. The hybrid simultaneously improves precision over Bi-LSTM-only and overall accuracy over both single-architecture baselines, confirming that long-range SSM modelling and bidirectional contextual encoding are complementary. All pairwise comparisons significant (p<0.001, McNemar’s test, Bonferroni-corrected).

**Table 9:** Performance of the SHAP-based verification system on the held-out test set.

Metric	Initial Model	After Feedback	Improvement
Accuracy	88.9%	90.8%	+1.9%
Precision	86.3%	**88.1%**	+1.8%
Recall	80.7%	**82.4%**	+1.7%
F1 Score	0.834	**0.852**	+0.017
AUC	0.8867	**0.9012**	+0.0145

The verification system employs a random forest classifier operating on the engineered feature set (Table 2). Its lower absolute performance relative to the primary discrimination system (Table 3) is expected given the simpler learning architecture; its primary function is independent cross-validation and SHAP-based interpretability rather than raw classification accuracy.

**Table 10:** SHAP-derived feature importance rankings before and after offline iterative recalibration (top 10).

Feature	Initial Rank	Post-Feedback Rank	Change
P/F ratio (oxygenation index)	1	1	+0
Ground-glass opacity area ratio	5	2	+3
PEEP value	2	3	−1
Plateau pressure	7	4	+3
Peak airway pressure	3	5	−2
Lung compliance	8	6	+2
Consolidation area ratio (bilateral)	6	7	−1
Tidal volume/predicted body weight	4	8	−4
Oxygenation index change rate	10	9	+1
Lactate value	9	10	−1

P/F ratio maintains the top rank throughout, consistent with the Berlin definition [1]. The rise of ground-glass opacity area (rank 5 → 2) and lung compliance (rank 8 → 6) after feedback reflects progressive re-weighting toward ARDS-specific imaging and mechanical features as the system accumulates high-confidence training examples. PEEP: positive end-expiratory pressure.

**Table 11:** Diagnostic consistency between the primary discrimination system and the verification system on the held-out test set (n=3742).

Agreement Category	Cases (*n*)	Proportion	Primary System Accuracy
High-confidence agreement (score >0.92)	2817	75.3%	92.8%
Low-confidence agreement (0.85< score ≤0.92)	567	15.2%	90.1%
Disagreement (score ≤0.85)	358	9.5%	87.4%
All cases	3742	100%	≈92.3%

Accuracy for disagreement cases is retrospectively evaluated against ground-truth labels from the dataset. The correlation between lower agreement scores and lower accuracy validates the clinical rationale for routing disagreement cases to physician review. Overall system accuracy of ≈92.3% is computed across all 3742 test cases.

**Table 12:** Performance gains attributable to dual-system collaboration via the confidence-gated offline refinement mechanism.

Metric	Primary System Gain	Verification System Gain
Accuracy	+2.6%	+1.9%
Precision	+2.4%	+1.8%
Recall	+4.5%	+1.7%
F1 Score	+0.035	+0.017

Gains represent the difference between post-recalibration and pre-recalibration performance (Table 3 and Table 9). The primary system benefits more from iterative recalibration than the verification system, reflecting its larger model capacity and its direct use of SHAP attribution maps for feature-selection gate modulation.

**Table 13:** Performance comparison with representative ARDS diagnostic methods.

Method	Reference	Dataset	Acc.	F1	AUC	Interpretability
Mamba-Bi-LSTM (internal)	This work	MIMIC-IV ^a^	92.8%	0.889	0.936	SHAP feature attribution
Mamba-Bi-LSTM (external)	This work	eICU ^a^	91.6%	0.871	0.921	SHAP feature attribution
Multimodal Transformer ^b^ (internal)	This work	MIMIC-IV	90.8%	0.865	0.924	Attention maps
Multimodal Transformer ^b^ (external)	This work	eICU (reduced-modality)	89.4%	0.850	0.910	Attention maps
LSTM-CNN ^c^	Meyer et al. [4]	MIMIC-III	88.4%	0.792	0.901	None
XGBoost + MIMIC-Extract	Suresh et al. [35]	MIMIC-III	86.3%	0.765	0.886	Feature importance
ResNet + EHR fusion	Jabbour et al. [5]	MIMIC-CXR/IV	87.9%	0.773	0.870	Grad-CAM
Attention-enhanced CNN	Das et al. [36]	eICU	85.7%	0.752	0.871	Attention heatmap
Multi-label deep learning	Jin et al. [37]	MIMIC-IV CXR	86.2%	0.765	0.863	Class activation map
Berlin criteria scoring	Wu et al. [38]	MIMIC-IV	82.3%	0.713	0.821	Rule-based paths

^a^ Internal: held-out MIMIC-IV test set (n=3742). External: eICU Collaborative Research Database (n=2594; 208 hospitals, US; no EEG modality). CXR: chest X-ray. All results report post-feedback performance. The performance drop from internal to external validation (Acc. −1.2%, AUC −0.015) demonstrates strong cross-institutional generalisability. ^b^ Multimodal Transformer: 4-layer encoder, 4 attention heads, $d_{\mathrm{model}}=64$, 7.0 M parameters; evaluated on the same held-out test set with identical preprocessing, modality configuration, and training protocol as the proposed framework. The performance gap (−2.0% accuracy, −0.012 AUC on MIMIC-IV internal) empirically justifies the Mamba-Bi-LSTM design: the Transformer incurs substantially higher latency (620 ms vs. 350 ms) and memory requirements (960 MB vs. 520 MB) without performance benefit, consistent with the known quadratic complexity of attention mechanisms on long clinical sequences. ^c^ Meyer et al. evaluated on post-cardiac surgery patients rather than ARDS specifically, reinforcing the need for ARDS-targeted architectures. All performance improvements are statistically significant (p<0.001, McNemar’s test, Bonferroni-corrected).

**Table 14:** Computational efficiency comparison for processing 24-h ARDS monitoring data.

Method	Inference Latency	Memory Usage	FLOPs	Concurrent Patients ^†^
Mamba-Bi-LSTM (ours)	350 ms	520 MB	4.5 G/s	≈164 pt/GPU/h
Multimodal Transformer (ours)	620 ms	960 MB	9.6 G/s	≈93 pt/GPU/h
LSTM-CNN [4]	712 ms	1370 MB	10.2 G/s	≈81 pt/GPU/h
XGBoost [35]	295 ms	430 MB	2.8 G/s	≈195 pt/GPU/h
ResNet-BiLSTM [5]	843 ms	1580 MB	12.6 G/s	≈68 pt/GPU/h
Attention-enhanced CNN [36]	675 ms	1250 MB	9.3 G/s	≈85 pt/GPU/h
Multi-label deep learning [37]	763 ms	1430 MB	11.5 G/s	≈75 pt/GPU/h
Berlin criteria scoring [38]	105 ms	180 MB	0.3 G/s	≈548 pt/GPU/h

All measurements performed on a single NVIDIA A100 40 GB GPU (CUDA 11.8). FLOPs: floating-point operations per second. ^†^ Throughput (concurrent ICU patients per GPU) is estimated as $\left\lfloor 57,400÷{\mathrm{latency}}_{\mathrm{ms}}\right\rfloor$, representing the maximum number of patients that can be concurrently monitored at approximately 1-minute assessment intervals (accounting for ∼5% system I/O overhead). The proposed system supports up to 164 concurrent patients per GPU under this protocol, versus 93 for the Multimodal Transformer baseline—sufficient for continuous real-time monitoring of a typical ICU with 10–30 beds. XGBoost is faster due to its shallow tree structure, but its accuracy and F1 are substantially lower (Table 13). The Berlin criteria rule-based system has the highest throughput but lowest diagnostic accuracy.

**Table 15:** Missing-modality ablation: accuracy and early warning time across four modality-availability configurations, with and without the missing-modality adaptation mechanism.

Configuration	With Mechanism		Without Mechanism		Gain
	Acc.	Warn.	Acc.	Warn.	
Full modality (EEG + Imaging)	92.8%	9.7 h	92.1%	9.4 h	+0.7%
EEG only (no Imaging)	91.7%	4.9 h	88.2%	4.8 h	+3.5%
Imaging only (no EEG)	92.0%	7.8 h	89.6%	7.5 h	+2.4%
No EEG, no Imaging	90.1%	4.1 h	87.4%	4.1 h	+2.7%

“With mechanism” denotes the full system with missing-modality adaptation (masking absent channels and redistributing modality-level weights). “Without mechanism” denotes naive input masking without weight redistribution. “No EEG, no Imaging” configuration is comparable to the eICU external validation setting (91.6%, Table 13), with the small discrepancy attributable to cross-institutional differences in the eICU cohort. Gain = accuracy difference between with and without mechanism. All comparisons significant (p<0.001, McNemar’s test).

## Data Availability

MIMIC-IV, MIMIC-III, and eICU Collaborative Research Database data are publicly available through PhysioNet (https://physionet.org) under credentialed access. Source code is available upon reasonable request to the corresponding author.

## References

[1] Ranieri V.M., Rubenfeld G.D., Taylor Thompson B., Ferguson N.D., Caldwell E., Fan E., Camporota L., Slutsky A.S. (2012). Acute respiratory distress syndrome: The Berlin Definition. JAMA J. Am. Med. Assoc..

[2] Churpek M.M., Yuen T.C., Winslow C., Meltzer D.O., Kattan M.W., Edelson D.P. (2016). Multicenter comparison of machine learning methods and conventional regression for predicting clinical deterioration on the wards. Crit. Care Med..

[3] Ruiz V.M., Goldsmith M.P., Shi L., Simpao A.F., Gálvez J.A., Naim M.Y., Nadkarni V., Gaynor J.W., Tsui F.R. (2022). Early prediction of clinical deterioration using data-driven machine-learning modeling of electronic health records. J. Thorac. Cardiovasc. Surg..

[4] Meyer A., Zverinski D., Pfahringer B., Kempfert J., Kuehne T., Sündermann S.H., Stamm C., Hofmann T., Falk V., Eickhoff C. (2018). Machine learning for real-time prediction of complications in critical care: A retrospective study. Lancet Respir. Med..

[5] Jabbour S., Fouhey D., Kazerooni E., Wiens J., Sjoding M.W. (2022). Combining chest X-rays and electronic health record (EHR) data using machine learning to diagnose acute respiratory failure. J. Am. Med. Inform. Assoc..

[6] Singhal L., Garg Y., Yang P., Tabaie A., Wong A.I., Mohammed A., Chinthala L., Kadaria D., Sodhi A., Holder A.L. (2021). eARDS: A multi-center validation of an interpretable machine learning algorithm of early onset Acute Respiratory Distress Syndrome (ARDS) among critically ill adults with COVID-19. PLoS ONE.

[7] Xiong Y., Gao Y., Qi Y., Zhi Y., Xu J., Wang K., Yang Q., Wang C., Zhao M., Meng X. (2025). Accuracy of artificial intelligence algorithms in predicting acute respiratory distress syndrome: A systematic review and meta-analysis. BMC Med. Inform. Decis. Mak..

[8] Gu A., Dao T. (2023). Mamba: Linear-time sequence modeling with selective state spaces. arXiv.

[9] Contreras M., Silva B., Shickel B., Davidson A., Ozrazgat-Baslanti T., Ren Y., Guan Z., Balch J., Zhang J., Bandyopadhyay S. (2024). APRICOT-Mamba: Acuity prediction in intensive care unit (ICU): Development and validation of a stability, transitions, and life-sustaining therapies prediction model. Res. Sq..

[10] Chen M., Xie J., Luo F., Ren Q. (2026). A Dual-System Approach for Epilepsy Diagnosis: Integrating Mamba-Bi-LSTM Architecture with SHAP-Based Verification. Biomed. Eng. Adv..

[11] Bommasani R., Klyman K., Longpre S., Kapoor S., Maslej N., Xiong B., Zhang D., Liang P. (2023). The foundation model transparency index. arXiv.

[12] Arrieta A.B., Díaz-Rodríguez N., Del Ser J., Bennetot A., Tabik S., Barbado A., García S., Gil-López S., Molina D., Benjamins R. (2020). Explainable Artificial Intelligence (XAI): Concepts, taxonomies, opportunities and challenges toward responsible AI. Inf. Fusion.

[13] Bermeo-Ovalle A., Naidech A.M. (2022). Can Early Electroencephalography Findings Predict Survival and Functional Outcome in Patients With Severe COVID-19 Infection?. Crit. Care Med..

[14] Johnson A.E., Bulgarelli L., Shen L., Gayles A., Shammout A., Horng S., Pollard T.J., Hao S., Moody B., Gow B. (2023). MIMIC-IV, a freely accessible electronic health record dataset. Sci. Data.

[15] Pollard T.J., Johnson A.E., Raffa J.D., Celi L.A., Mark R.G., Badawi O. (2018). The eICU Collaborative Research Database, a freely available multi-center database for critical care research. Sci. Data.

[16] Mascia L., D’Albo R., Cavalli I., Giaccari L., Della Giovampaola M., Donati B. (2025). Organ crosstalk: Brain-lung interaction. Front. Med..

[17] Park H., Lee C.H. (2024). The impact of pulmonary disorders on neurological health (lung-brain axis). Immune Netw..

[18] Mikkelsen M.E., Christie J.D., Lanken P.N., Biester R.C., Thompson B.T., Bellamy S.L., Localio A.R., Demissie E., Hopkins R.O., Angus D.C. (2012). The adult respiratory distress syndrome cognitive outcomes study: Long-term neuropsychological function in survivors of acute lung injury. Am. J. Respir. Crit. Care Med..

[19] Pasini E., Bisulli F., Volpi L., Minardi I., Tappatà M., Muccioli L., Pensato U., Riguzzi P., Tinuper P., Michelucci R. (2020). EEG findings in COVID-19 related encephalopathy. Clin. Neurophysiol..

[20] Kubota T., Gajera P.K., Kuroda N. (2021). Meta-analysis of EEG findings in patients with COVID-19. Epilepsy Behav..

[21] Newcombe V.F., Dangayach N.S., Sonneville R. (2021). Neurological complications of COVID-19. Intensive Care Med..

[22] Cleveland W.S., Devlin S.J. (1988). Locally weighted regression: An approach to regression analysis by local fitting. J. Am. Stat. Assoc..

[23] Ronneberger O., Fischer P., Brox T. (2015). U-net: Convolutional networks for biomedical image segmentation. Proceedings of the International Conference on Medical Image Computing and Computer-Assisted Intervention.

[24] Hochreiter S., Schmidhuber J. (1997). Long short-term memory. Neural Comput..

[25] DeLong E.R., DeLong D.M., Clarke-Pearson D.L. (1988). Comparing the areas under two or more correlated receiver operating characteristic curves: A nonparametric approach. Biometrics.

[26] HH J. (1958). The ten-twenty electrode system of the international federation. Electroenceph. Clin. Neurophysiol..

[27] Kipf T.N., Welling M. (2016). Semi-supervised classification with graph convolutional networks. arXiv.

[28] Subasi A. (2007). EEG signal classification using wavelet feature extraction and a mixture of expert model. Expert Syst. Appl..

[29] Acharya U.R., Oh S.L., Hagiwara Y., Tan J.H., Adam M., Gertych A., San Tan R. (2017). A deep convolutional neural network model to classify heartbeats. Comput. Biol. Med..

[30] Lundberg S.M., Lee S.I. (2017). A unified approach to interpreting model predictions. Adv. Neural Inf. Process. Syst..

[31] Lundberg S.M., Erion G., Chen H., DeGrave A., Prutkin J.M., Nair B., Katz R., Himmelfarb J., Bansal N., Lee S.I. (2020). From local explanations to global understanding with explainable AI for trees. Nat. Mach. Intell..

[32] Bengio Y., Louradour J., Collobert R., Weston J. (2009). Curriculum learning. Proceedings of the 26th Annual International Conference on Machine Learning.

[33] Nath M.K., Mookkandi K., Balakrishnan K., Mishra M., Kavitha M., Mathumathi M. (2026). Bottleneck of AI driven techniques for identifying indian agricultural diseases: A systematic review and future directives. Results Eng..

[34] Nath M.K., Dash S.S., Mookkandi K., A. T. (2026). Towards intelligent phonocardiogram analysis: Advances and future perspectives in cardiovascular disease detection. Eng. Appl. Artif. Intell..

[35] Suresh H., Hunt N., Johnson A., Celi L.A., Szolovits P., Ghassemi M. (2017). Clinical intervention prediction and understanding using deep networks. arXiv.

[36] Das P.P., Wiese L., Mast M., Böhnke J., Wulff A., Marschollek M., Bode L., Rathert H., Jack T., Schamer S. (2025). An attention-based bidirectional LSTM-CNN architecture for the early prediction of sepsis. Int. J. Data Sci. Anal..

[37] Jin Y., Lu H., Zhu W., Huo W. (2023). Deep learning based classification of multi-label chest X-ray images via dual-weighted metric loss. Comput. Biol. Med..

[38] Wu J., Liu C., Xie L., Li X., Xiao K., Xie G., Xie F. (2022). Early prediction of moderate-to-severe condition of inhalation-induced acute respiratory distress syndrome via interpretable machine learning. BMC Pulm. Med..

[39] Cil A.E., Buyuktanir T., Yildiz K. (2026). Comprehensive explainable AI approach for audit opinion classification using feed-forward neural networks. Knowl.-Based Syst..

[40] Baspinar U., Selcuk Varol H., Yildiz K. (2012). Classification of hand movements by using artificial neural network. 2012 International Symposium on Innovations in Intelligent Systems and Applications.

